# Development of a genetically tailored implantation hepatocellular carcinoma model in Oncopigs by somatic cell CRISPR editing

**DOI:** 10.1242/dmm.052079

**Published:** 2025-01-29

**Authors:** Lobna Elkhadragy, Maximillian J. Carlino, Luke R. Jordan, Thomas Pennix, Nahed Ismail, Grace Guzman, Jonathan P. Samuelson, Lawrence B. Schook, Kyle M. Schachtschneider, Ron C. Gaba

**Affiliations:** ^1^ Department of Radiology, University of Illinois at Chicago, Chicago, IL 60612, USA; ^2^ Department of Pathology, University of Illinois at Chicago, Chicago, IL 60612, USA; ^3^ Department of Veterinary Clinical Medicine, University of Illinois at Urbana-Champaign, Champaign, IL 61802, USA; ^4^ Department of Animal Sciences, University of Illinois at Urbana-Champaign, Champaign, IL 61801, USA

**Keywords:** HCC model, Porcine model, Large-animal model, CRISPR gene editing, Precision models

## Abstract

Hepatocellular carcinoma (HCC) is an aggressive disease with poor prognosis, necessitating preclinical models for evaluating novel therapies. Large-animal models are particularly valuable for assessing locoregional therapies, which are widely employed across HCC stages. This study aimed to develop a large-animal HCC model with tailored tumor mutations. The Oncopig, a genetically engineered pig with inducible *TP53^R167H^* and *KRAS^G12D^*, was used in the study. Hepatocytes were isolated from Oncopigs and exposed to Cre recombinase *in vitro* to create HCC cells, and additional mutations were introduced by CRISPR/Cas9 knockout of *PTEN* and *CDKN2A*. These edits increased Oncopig HCC cell proliferation and migration. Autologous HCC cells with these CRISPR edits were implanted into Oncopigs using two approaches: ultrasound-guided percutaneous liver injections, which resulted in the development of localized intrahepatic masses, and portal vein injections, which led to multifocal tumors that regressed over time. Tumors developed by both approaches harbored *PTEN* and *CDKN2A* knockout mutations. This study demonstrates the feasibility of developing genetically tailored HCC tumors in Oncopigs using somatic cell CRISPR editing and autologous implantation, providing a valuable large-animal model for *in vivo* therapeutic assessment.

## Teaser

**Summary:** We demonstrate the feasibility of developing genetically tailored hepatocellular carcinoma tumors in transgenic pigs by somatic cell CRISPR editing and autologous orthotopic implantation, providing a valuable large-animal model for *in vivo* assessment of therapeutic strategies.

## INTRODUCTION

Liver cancer is a major global health burden, ranking as the sixth most common cancer and the third leading cause of cancer-related mortality (Sung et al., 2021). The most common type of liver cancer is hepatocellular carcinoma (HCC), accounting for 75-85% of cases (Sung et al., 2021). Major risk factors for HCC include chronic viral hepatitis, chronic alcohol consumption, diabetes and metabolic syndrome, all of which contribute to chronic liver disease, or cirrhosis (Llovet et al., 2021b). Patients with early-stage HCC are eligible for curative surgical procedures such as resection or transplantation. However, most HCC patients are diagnosed at intermediate or advanced stages, for which the standard of care treatment is locoregional therapy (LRT) or systemic therapy, respectively (Llovet et al., 2021b). LRTs, such as ablation and arterial therapies, are minimally invasive, image-guided procedures employed in 50-60% of HCC cases (Llovet et al., 2021a). Approved systemic therapies for HCC include antiangiogenic tyrosine kinase inhibitors and immune checkpoint inhibitors (Llovet et al., 2021b). Although the median overall survival exceeds 5 years for early-stage HCC, it is 25-30 months for intermediate-stage HCC and 10-19 months for advanced-stage HCC (Llovet et al., 2021a). This highlights the need for more effective therapeutic strategies for the management of unresectable HCC.

Advances in genome sequencing technologies have greatly advanced our knowledge about the mutational landscape of HCC (The Cancer Genome Atlas Research Network, 2017; Chaisaingmongkol et al., 2017; Fujimoto et al., 2016; Boyault et al., 2007; Kaseb et al., 2019; von Felden et al., 2021). Frequently mutated genes in HCC include *TERT*, *TP53* and *CTNNB1*. Over half of HCC cases exhibit somatic tumor inactivation of cyclin-dependent kinase inhibitor 2A (*CDKN2A*), the gene that encodes p16^INK4A^ and p14^ARF^ proteins, owing to epigenetic silencing, loss-of-function mutations or chromosomal deletion (The Cancer Genome Atlas Research Network, 2017). The tumor suppressor gene *PTEN* is mutated or deleted in ∼7-10% of HCC cases (The Cancer Genome Atlas Research Network, 2017; Fujimoto et al., 2016; Guichard et al., 2012). The comprehensive analysis of HCC genetic alterations has laid the foundation for the development of precision medicine approaches targeting these molecular aberrations. Precision medicine can be delivered systemically and/or locally into tumors by LRTs, presenting a promising therapeutic strategy for unresectable HCC (Llovet et al., 2021a; Nault et al., 2020; Rebouissou and Nault, 2020; Harding et al., 2019).

Testing and validation of novel therapeutics necessitates the availability of preclinical models with precise tumor mutations that recapitulate human HCC. Although murine models are valuable, they have limitations that impede successful translation of findings to clinical trials. A major limitation is the vast difference in metabolic rate and drug metabolism between mice and humans, which makes mouse models poor models of toxicity, sensitivity and efficacy in preclinical drug studies (He et al., 2015; Heindryckx et al., 2009). Another major limitation of mouse models is their small size, which prohibits testing of LRTs and other device-based techniques employed in clinical practice (Aravalli et al., 2009). Currently, the rabbit VX2 model is the most widely used model for testing liver-directed LRTs. However, the VX2 model has significant drawbacks, including tumor squamous cell origin and biological dissimilarity with HCC (Aravalli et al., 2009; Kidd and Rous, 1940; Parvinian et al., 2014). Therefore, there is a critical need for large-animal models of HCC with clinically relevant tumor mutations to improve the translation of novel precision therapeutics to clinical practice.

Our study addresses this necessity by developing a feasible approach using somatic cell CRISPR gene editing and a transgenic pig, the Oncopig. Pigs are valuable models for human diseases owing to their similarities to humans in size, physiology, anatomy, genetics and immunity (Flisikowska et al., 2016; Groenen et al., 2012). Importantly, the pig's basal metabolic rate and xenosensor pregnane X receptor, responsible for the metabolism of half of all prescription drugs, are very similar to those of humans, increasing the translatability of preclinical studies (Gray et al., 2010; Pollock et al., 2007; Zuber et al., 2002). The Oncopig is a genetically engineered pig model with Cre recombinase-inducible expression of *KRAS^G12D^* and *TP53^R167H^* (Schook et al., 2015). Leveraging CRISPR editing, the Oncopig and image-guided procedures, we demonstrate the feasibility of creation of localized and multifocal HCC tumors harboring specific tumor mutations. Although *PTEN* and *CDKN2A* knockout (KO) mutations were induced in this study owing to their clinical relevance, this modeling platform is adaptable for developing Oncopig HCC models with diverse gene alterations. These novel models are invaluable tools for evaluating the efficacy of innovative LRTs and/or systemic therapies tailored to distinct HCC gene alterations, thus advancing our understanding and treatment of this challenging disease.

## RESULTS

### CRISPR KO of *PTEN* and *CDKN2A* in Oncopig HCC cells models functional effects in human HCC

**Fig. 1.**
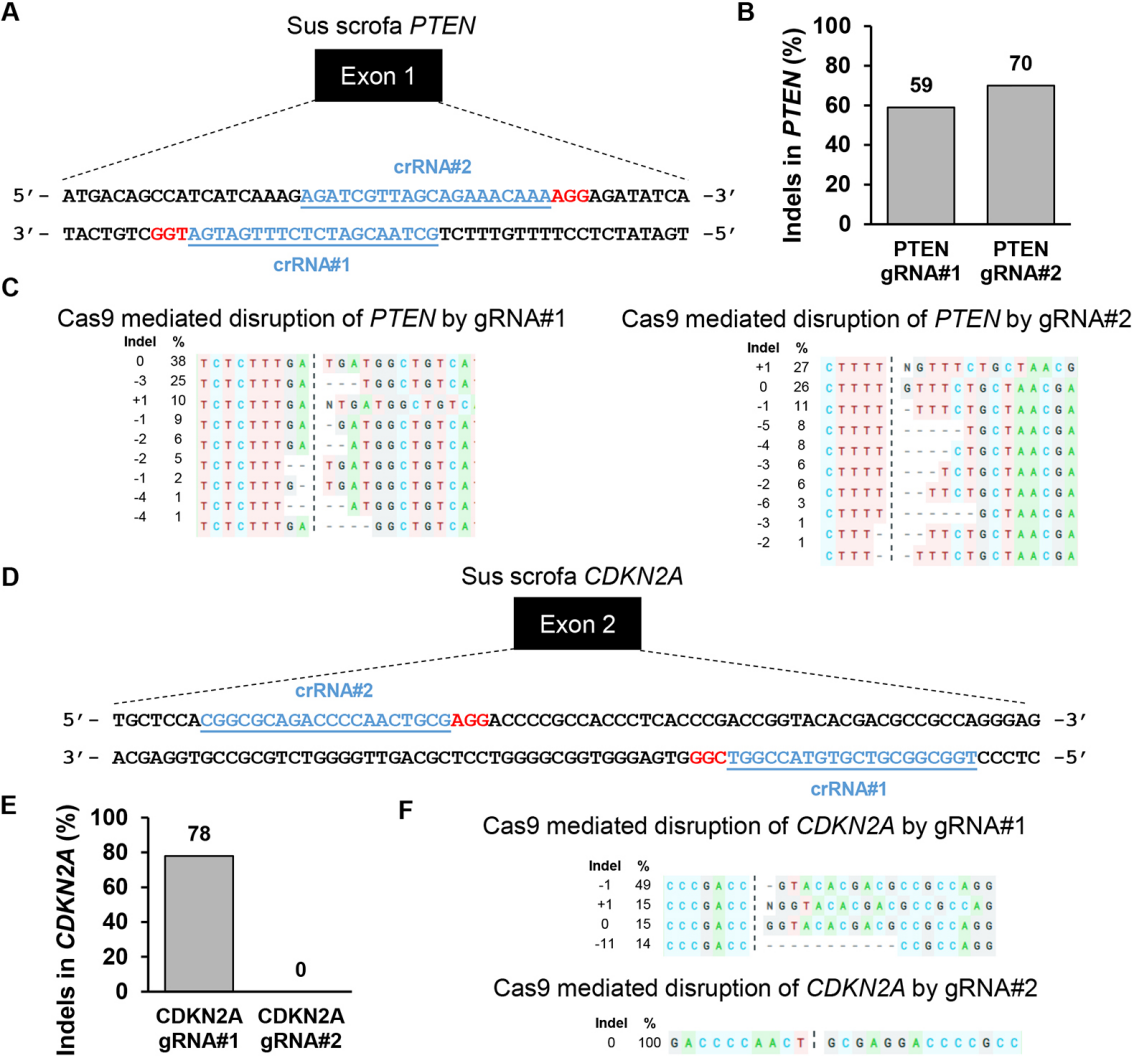
**Screening and validation of CRISPR gRNAs targeting porcine *PTEN* and *CDKN2A* genes.** (A) Schematic representation of porcine *PTEN* locus showing the location of spacer sequences crRNA#1 and crRNA#2 (blue underlined font). Protospacer adjacent motif (PAM) sequences are marked in red. (B) CRISPR/Cas9 editing efficiency of two individual *PTEN*-targeting gRNAs. Porcine A343 hepatocellular carcinoma (HCC) cells were transfected with ribonucleoproteins (RNPs) comprising Cas9 and each gRNA. Genomic DNA was collected 2 days post-transfection and analyzed by Sanger sequencing–ICE analysis. The bar graph depicts the percentages (%) of total reads that displayed indels at the gRNA target site occurring as a result of non-homologous end joining. (C) Type and frequency of *PTEN* indels mapped to the reference sequence. Dashed lines indicate the predicted Cas9 cleavage position; dashes indicate deleted bases. N, nucleotide insertion. (D) Schematic representation of porcine *CDKN2A* locus showing the location of spacer sequences crRNA#1 and crRNA#2 (blue underlined font). PAM sequences are marked in red. (E) CRISPR/Cas9 editing efficiency of two individual *CDKN2A*-targeting gRNAs. Porcine A343 HCC cells were transfected with RNPs comprising Cas9 and each gRNA. Genomic DNA was collected 2 days post-transfection and analyzed by Sanger sequencing–ICE analysis. The bar graph depicts the % of total reads that displayed indels at the gRNA target site. (F) Type and frequency of *CDKN2A* indels mapped to the reference sequence. Dashed lines indicate the predicted Cas9 cleavage position; dashes indicate deleted bases. N, nucleotide insertion.

Oncopig HCC cells were developed by *in vitro* exposure of isolated hepatocytes to Cre recombinase as previously described (Elkhadragy et al., 2020; Gaba et al., 2020; Schachtschneider et al., 2017). To induce loss-of-function mutations in porcine *PTEN* and *CDKN2A* mimicking frequently observed human HCC genetic alterations, two individual CRISPR guide RNAs (gRNAs) against each gene were designed and screened in Oncopig HCC cells (Fig. 1). Successful targeting resulted in small indels around the predicted Cas9 cleavage sites. Among the tested gRNAs, *PTEN* gRNA#2 and *CDKN2A* gRNA#1 demonstrated higher editing efficiencies and hence were used in the subsequent experiments.

**Fig. 2.**
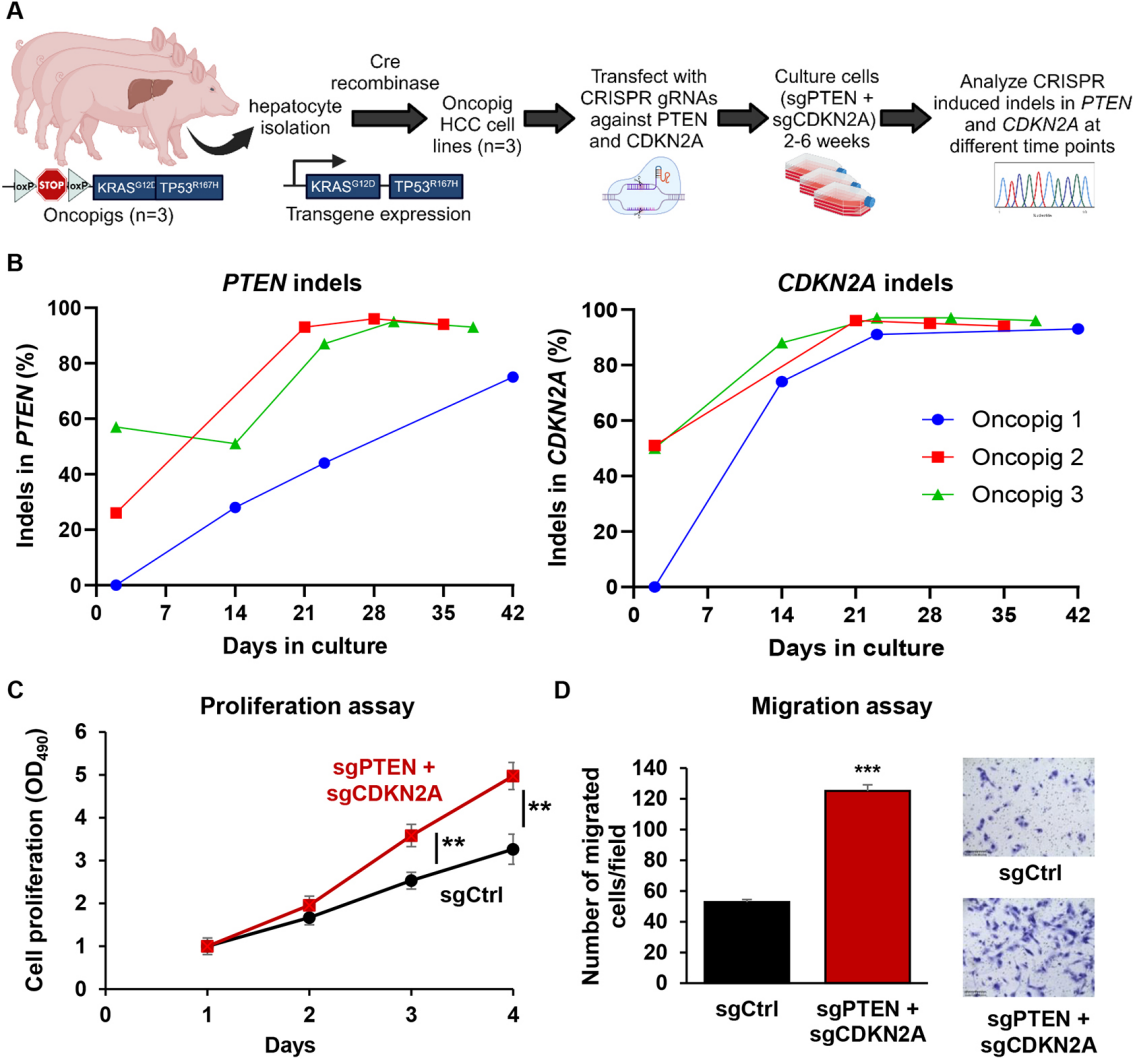
**Depletion of *PTEN* and *CDKN2A* confers growth advantage and increases Oncopig HCC cell proliferation and migration.** (A) Schematic representation of the experiment. Porcine HCC cells were developed from three individual Oncopigs and transfected with gRNAs against *PTEN* and *CDKN2A* simultaneously. Cells were passaged, and DNA was obtained at several time points for sequencing analysis of CRISPR-induced indels. Created in BioRender by Elkhadragy, L., 2025. https://BioRender.com/b48t314. This figure was sublicensed under CC-BY 4.0 terms. (B) Enrichment of cells with *PTEN* and *CDKN2A* knockout over time. Percentage indels in *PTEN* (left) and *CDKN2A* (right) over time in culture is shown in the graphs. (C) Cell proliferation was determined by MTS assay for Oncopig HCC cells transfected with control non-targeting gRNA (sgCtrl) or gRNAs targeting *PTEN* and *CDKN2A* (sgPTEN+sgCDKN2A). Cell viability at different time points was measured and expressed as optical density at 490 nm (OD_490_) normalized to values on day 1. Statistical analysis was conducted to compare the viability of the cells at each time point by two-way ANOVA. **P<0.005. (D) Migration of Oncopig HCC sgPTEN+sgCDKN2A cells in comparison to sgCtrl cells was assessed by transwell cell migration assay. Quantitated migration ability is presented as the number of migrated cells per field. Values in the bar graph represent mean±s.e. (*n*=8 fields). ***P<0.0001. Representative images of migrated cells stained with Crystal Violet are shown on the right. Scale bars: 250 μm.

To assess the functional effects of *PTEN* and *CDKN2A* depletion in Oncopig HCC cells, *PTEN* and *CDKN2A* gRNAs were transfected simultaneously into each of three HCC cell lines developed from Oncopigs 1, 2 and 3 (Fig. 2A). Analysis of *PTEN* and *CDKN2A* indels in the cultured cells at multiple time points revealed a progressive increase in the frequency of frameshift mutations in both genes over time (Fig. 2B; Figs S1-S3). By 4-6 weeks post-transfection, the HCC cells were predominantly enriched with frameshift mutations in both *PTEN* and *CDKN2A*, and these cells were referred to as sgPTEN+sgCDKN2A. This enrichment indicates that PTEN and CDKN2A deficiencies confer a selective growth advantage to Oncopig HCC cells. Compared to control Oncopig HCC cells transfected with a non-targeting gRNA, sgPTEN+sgCDKN2A cells exhibited increased proliferation and migration (Fig. 2C,D). These findings underscore the functional significance of PTEN and CDKN2A deficiencies in conferring selective growth advantage and promoting proliferation and migration in Oncopig HCC cells, consistent with their established tumor suppressor roles in human HCC.

### Development of Oncopig intrahepatic tumors by percutaneous injection of autologous HCC cells

**Fig. 3.**
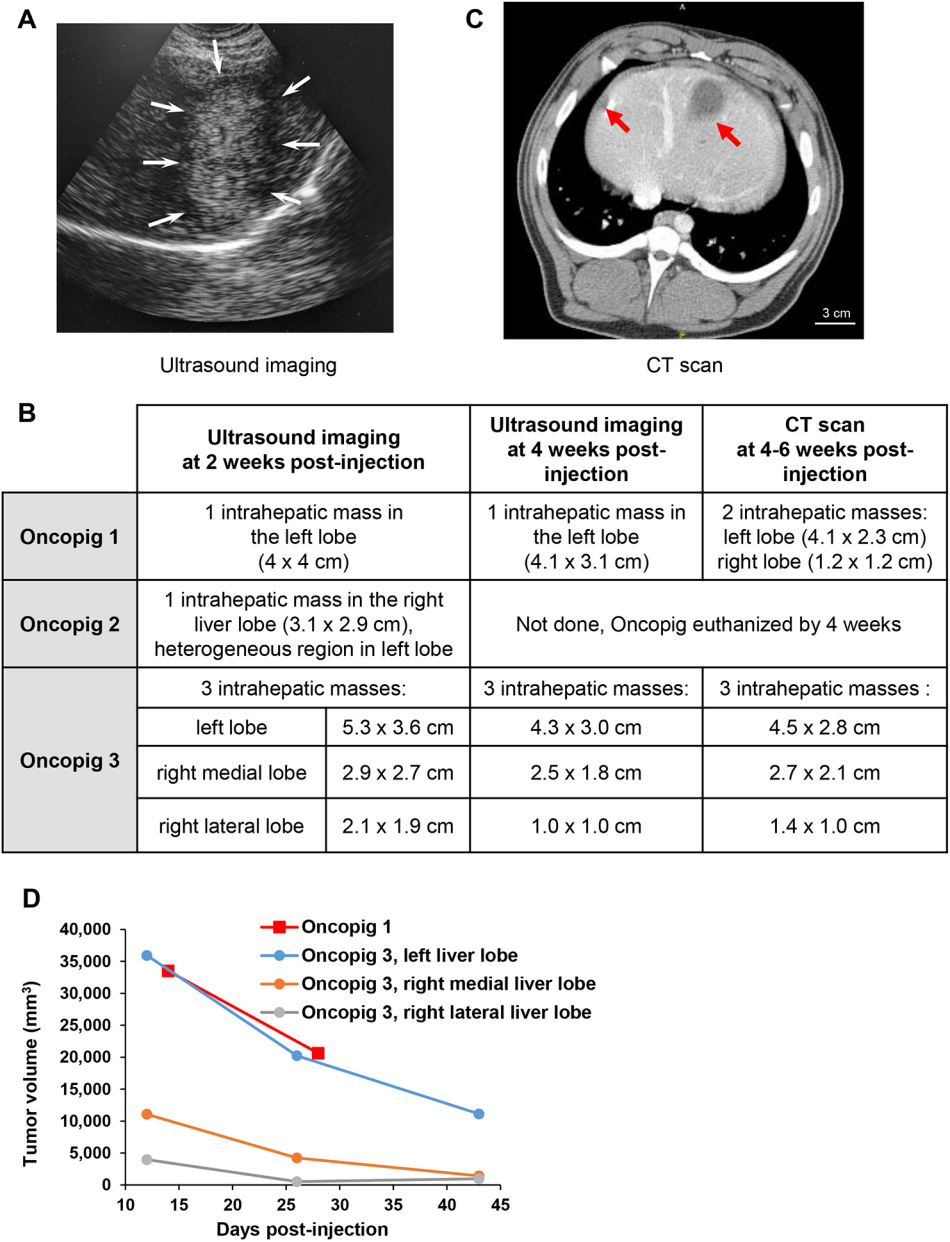
**Development and imaging of Oncopig intrahepatic tumors induced by percutaneous injection of autologous CRISPR-edited cells.** (A) Representative ultrasound image of an intrahepatic tumor developed in the left liver lobe of Oncopig 3 at 2 weeks post-injection. White arrows indicate the tumor edges. (B) Monitoring of intrahepatic tumor development in Oncopigs following the injection of autologous CRISPR-edited cells by ultrasound imaging and computed tomography (CT). Ultrasound imaging was done at 2 and 4 weeks post-injection. CT was done at 6 weeks post-injection in Oncopig 1 and at 4 weeks post-injection in Oncopig 3. Sizes of tumors detected by imaging are shown in the table. (C) Representative CT scan depicting two intrahepatic tumors (red arrows) in Oncopig 3 at 4 weeks post-injection. Scale bar: 3 cm. (D) Graphical representation of tumor volume (mm^3^) plotted against days post-injection.

To develop a clinically relevant human-size model with genetically tailored localized HCC tumors, an orthotopic implantation approach was used in the Oncopigs. Owing to the outbred nature of pigs, an autologous cell injection approach was utilized. HCC cells with CRISPR-induced *PTEN* and *CDKN2A* KO developed from Oncopigs 1, 2 and 3 as described above were injected autologously into four sites of the liver parenchyma under ultrasound guidance. Prior to cell implantation, alcoholic liver fibrosis was induced in the three Oncopigs to mimic the fibrotic liver microenvironment common in human HCC. Liver fibrosis was induced by intrahepatic injection of an ethanol–ethiodized oil mixture (Gaba et al., 2018) and oral alcohol administration (Table 1). Imaging assessments confirmed the development of intrahepatic masses in all three Oncopigs (Fig. 3).

**Table 1.** Animals and experimental procedures

Animal number	Oral alcohol solution administration	Intrahepatic injection of ethanol–ethiodized oil	Cell injection approach	Imaging assessment timing post-injection	Euthanasia time post-injection	Tumors detected by imaging?	Cancer on microscopy?
Oncopig 1	1 week before cell injection, until euthanasia	Simultaneous with cell injections	Ultrasound-guided injection of 60 million cells into each of four sites in the liver parenchyma	US: 2 and 4 weeks CT: 6 weeks	2.5 months	Yes	Yes
Oncopig 2	6 weeks before cell injections, until euthanasia	2 weeks before cell injections		US: 2 weeks	4 weeks	Yes	Yes
Oncopig 3	6 weeks before cell injections, until euthanasia	2 weeks before cell injections		US: 2 and 4 weeks CT: 4 weeks	3 months	Yes	Yes
Oncopig 4	–	–	Portal vein injection of 150 million cells	–	9 days	Imaging revealed heterogeneous liver parenchyma and portal vein thrombosis	Yes
Oncopig 5	–	–		US: 2 weeks	3 weeks		Yes
Oncopig 6	5 months before cell injections, for a total of 5.5 months	4.6 months before cell injections	Portal vein injection of 70 million cells	US: 1 week CT: 6 and 11 weeks	2.75 months	Yes, but regressed at later time point	No
Oncopig 7	4.4 months before cell injections, for a total of 5.5 months	4.1 months before cell injections		US: 1 week CT: 7 weeks and 4 months	4 months	No	No

The table includes a summary of cell injection approaches, liver fibrosis induction approaches, and imaging and histological outcomes for the pigs included in the study. CT, computed tomography; US, ultrasound.

Ultrasound imaging at 2 weeks post-injection revealed one intrahepatic mass in each of Oncopigs 1 and 2, and three masses in Oncopig 3, with varying imaging characteristics (hypoechoic, hyperechoic or heterogeneous) (Fig. 3A,B). By week 4, the masses were consistently detected in Oncopigs 1 and 3 by ultrasound imaging, with an observed size reduction compared to earlier measurements (Fig. 3B,D). Computed tomography (CT) scans were performed at 4-6 weeks post-injection to provide more sensitive detection of the tumors. In Oncopig 1, CT scan at 6 weeks post-injection identified two masses, while in Oncopig 3, CT scan at 4 weeks post-injection detected three masses, consistent with the ultrasound findings (Fig. 3B,C). These results demonstrate the successful development and imaging of Oncopig intrahepatic HCC tumors using clinically relevant imaging modalities.

Despite successful tumor development in Oncopigs 1 and 3 without major adverse events or blood test alterations (Fig. S4), Oncopig 2 experienced procedure-related adverse events. At 2 weeks post-injection, ultrasound imaging in Oncopig 2 revealed gallbladder sludge and an undefined heterogeneous region in the left liver lobe. Histological analysis of a biopsy from this region showed bile duct proliferation, arterial vascular proliferation and fibrous tissue (Fig. S5A). By 4 weeks post-injection, Oncopig 2 exhibited clinical deterioration, including lethargy, loss of appetite and jaundice, necessitating euthanasia. Necropsy revealed excessive bile flow and necrotic liver regions (Fig. S5B), and histological analysis showed cholestasis, high-stage fibrosis and infarcted liver tissue, indicative of severe liver damage (Fig. S5C). Serum analysis demonstrated marked elevations in total bilirubin, gamma-glutamyl transferase, alkaline phosphatase and aspartate transferase levels in this Oncopig compared to those in normal age-matched Oncopigs (Fig. S4). These findings suggest the development of biliary stricture in Oncopig 2, potentially due to bile duct injury during percutaneous injections or obstruction of the gall bladder by a growing mass.

### Histological and next-generation sequencing (NGS) analyses confirm the development of genetically tailored HCC in Oncopigs

**Fig. 4.**
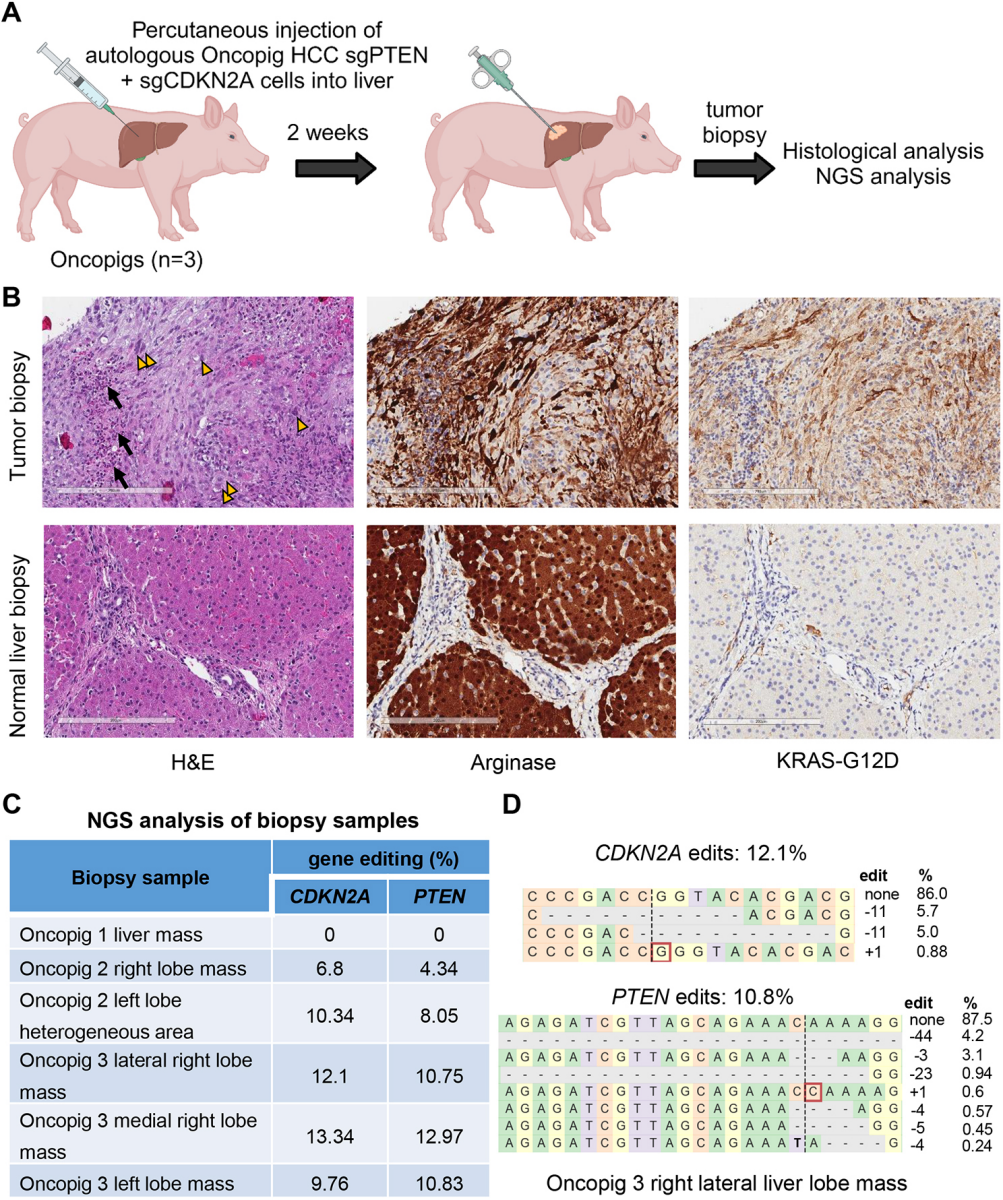
**Histological and next-generation sequencing (NGS) analyses of tumor biopsies confirm the development of HCC tumors with *PTEN* and *CDKN2A* disruption.** (A) Ultrasound-guided biopsies were obtained from intrahepatic tumors and normal uninvolved liver at 2 weeks post-injection for histological and NGS analyses. Created in BioRender by Elkhadragy, L., 2025. https://BioRender.com/g69j541. This figure was sublicensed under CC-BY 4.0 terms. (B) Representative images of tumor or normal liver biopsies stained with Hematoxylin and Eosin (H&E), and for arginase-1 or KRAS^G12D^. Neoplastic cells in tumor biopsies displayed atypical morphology and cytological abnormalities (yellow arrowheads) and were infiltrated by immune cells (black arrows). Neoplastic cells showed positive arginase-1 and KRAS^G12D^ immunohistochemical staining (brown staining). Normal liver biopsies show typical lobulated liver architecture with mild fibrosis. Hepatocytes in normal liver show positive arginase-1 staining and negative KRAS^G12D^ staining. Scale bars: 200 μm. (C) Targeted NGS analysis detects *PTEN* and *CDKN2A* edits in Oncopig intrahepatic tumor biopsy samples. (D) Reads detected by targeted NGS analysis in an Oncopig intrahepatic tumor mapped to the reference sequences. The percentages of reads of each sequence are shown on the right. Dashed lines indicate the predicted Cas9 cleavage position; red boxes show insertions; dashes indicate deleted bases.

The development of genetically tailored HCC in Oncopigs was confirmed through histological and NGS analyses of tumor biopsies collected at 2 weeks post-injection (Fig. 4). Given the large size of pigs, it is possible to obtain liver and tumor biopsies under ultrasound guidance, enabling tumor analysis at multiple time points without increasing animal use. Analysis of formalin fixed paraffin-embedded tumor biopsy samples by Hematoxylin and Eosin (H&E) staining showed neoplastic cells with atypical morphology and cytological abnormalities infiltrated with immune cells, whereas normal liver biopsies displayed typical lobulated liver architecture with mild fibrosis (Fig. 4B). Immunohistochemical (IHC) staining for KRAS^G12D^ confirmed that the neoplastic cells were the injected cells expressing the transgene. Arginase-1 staining validated these as HCC cells (Fujiwara et al., 2012; Larson et al., 2024; McKnight et al., 2012). As expected, normal liver tissue exhibited positive arginase-1 staining and negative KRAS^G12D^ staining (Fig. 4B).

To analyze the CRISPR-induced edits in tumor biopsy samples, targeted NGS was performed rather than Sanger sequencing because tumor cells were expected to represent a small fraction of each biopsy sample, necessitating a more sensitive sequencing technique. NGS analysis of the tumor biopsies confirmed the presence of CRISPR-induced edits in *PTEN* and *CDKN2A* in Oncopigs 2 and 3 (Fig. 4C,D; Fig. S6), indicating successful development of genetically tailored HCC in Oncopigs. The percentage of *PTEN* and *CDKN2A* edits in the biopsy samples was nearly 10%, reflecting the presence of other non-tumor cells in the samples, including normal liver tissue adjacent to the tumors and tumor-infiltrating immune cells. However, the biopsy from the tumor in Oncopig 1 did not harbor *PTEN* or *CDKN2A* gene edits, possibly owing to sampling bias of the heterogeneous mass, which showed KRAS^G12D^ IHC staining in a different biopsy from the same mass. The variability between biopsies from the same mass and the small overall amount of KRAS^G12D^-positive cells per biopsy is notable in low-magnification representative biopsy images (Fig. S7). Interestingly, the heterogeneous region in the left liver lobe of Oncopig 2 also harbored *PTEN* and *CDKN2A* gene edits (Fig. S6), suggesting the development of a diffuse, undefined lesion rather than a well-defined tumor at this injection site. As expected, adjacent normal liver biopsies did not harbor *PTEN* or *CDKN2A* gene edits, confirming the specificity of the genetic alterations to the tumor cells subjected to *in vitro* CRISPR/Cas9. Together, these results demonstrate the development of genetically tailored HCC tumors in Oncopigs by autologous implantation within 2 weeks.

### Oncopig intrahepatic tumors exhibit a strong immune response and central necrosis at 10-12 weeks post-implantation

**Fig. 5.**
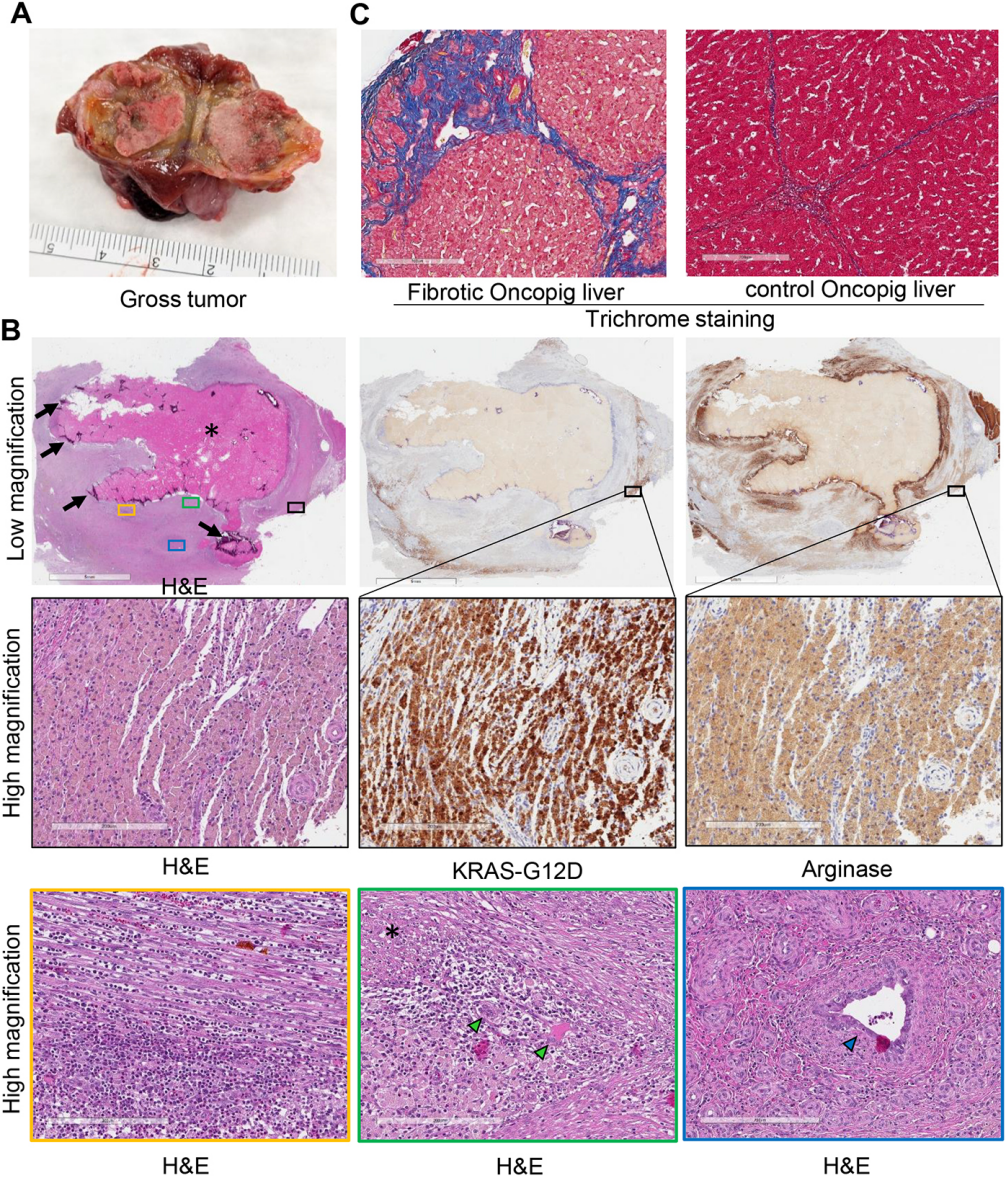
**Tumors exhibit a strong immune response and necrotic center at 10-12 weeks post-injection of autologous HCC cells.** (A) Representative image of gross tumor harvested from Oncopig 1 at 10** **weeks post-injection of autologous CRISPR-edited HCC cells. (B) Representative microscopy images of tumor sections stained with H&E, and for arginase-1 and KRAS^G12D^. Tumors displayed a large necrotic center (marked by asterisk) with peripheral calcification (black arrows) and areas with a dense inflammatory response. Neoplastic cells formed sheets and displayed variably distinct cell margins, moderate eosinophilic cytoplasm, and stained positively for arginase-1 and KRAS^G12D^ (insets with black border). The inset with a yellow border shows immune cells, including neutrophils, eosinophils, lymphocytes and plasma cells interdigitated between strands of fibrin. The inset with a green border shows multinucleated giant cells (green arrowheads) surrounded by mixed cell inflammatory infiltrate, including lymphocytes, histiocytes and plasma cells surrounded by early organizing fibrosis. A necrotic region is marked by an asterisk. The inset with a blue border shows a bile duct (blue arrowhead) surrounded by blood vessels. Scale bars: 5 mm and 200 μm (insets). (C) Trichrome staining of normal uninvolved liver from Oncopig 1 highlights collagen as blue and depicts liver fibrosis. Trichrome staining of liver section from an age-matched control Oncopig is shown on the right. Scale bars: 200 μm.

Upon euthanasia of Oncopigs 1 and 3 at 10 or 12 weeks post-implantation, respectively, three liver masses were identified and harvested from each Oncopig (Fig. 5A). For Oncopig 3, the imaging results were consistent with necropsy findings, as the three masses detected by imaging were found and harvested from their expected locations. For Oncopig 1, three masses were found during necropsy, although only two masses were detected by CT scan, likely due to the small size of the third mass. Histological analysis of these masses revealed that they had large necrotic centers surrounded at the periphery by a thin sheet of neoplastic cells and dense inflammatory regions (Fig. 5B). The neoplastic cells displayed variably distinct cell margins, moderate eosinophilic cytoplasm, and round to oval nuclei containing coarse chromatin and central nucleoli. These cells stained positively for both arginase-1 and KRAS^G12D^. Immune cells, including lymphocytes and giant cells, were present, indicating a strong immune response within the tumor microenvironment compared to uninvolved pig liver. Peripheral tumor areas included bile duct elements, blood and fibrin. Acellular material, likely to be remnants of gelatin sponge used as a scaffold for cell injection, was observed in some masses. Also, some masses were lined by calcification and fibrotic liver tissue. Consistent with liver fibrosis induction performed in these Oncopigs, histological analysis of liver tissues stained with H&E and trichrome revealed fibrosis (Fig. 5C). NGS analysis did not detect *PTEN* or *CDKN2A* edits in any of these six masses harvested at euthanasia, likely due to the very small percentage and peripheral localization of viable tumor samples as observed by IHC staining (Fig. 5B). These findings demonstrate the occurrence of necrosis and strong immune response in orthotopic tumors developed in Oncopigs 10-12 weeks after autologous cell implantation.

### Development of multifocal HCC in Oncopigs by injection of autologous cells through the portal vein

**Fig. 6.**
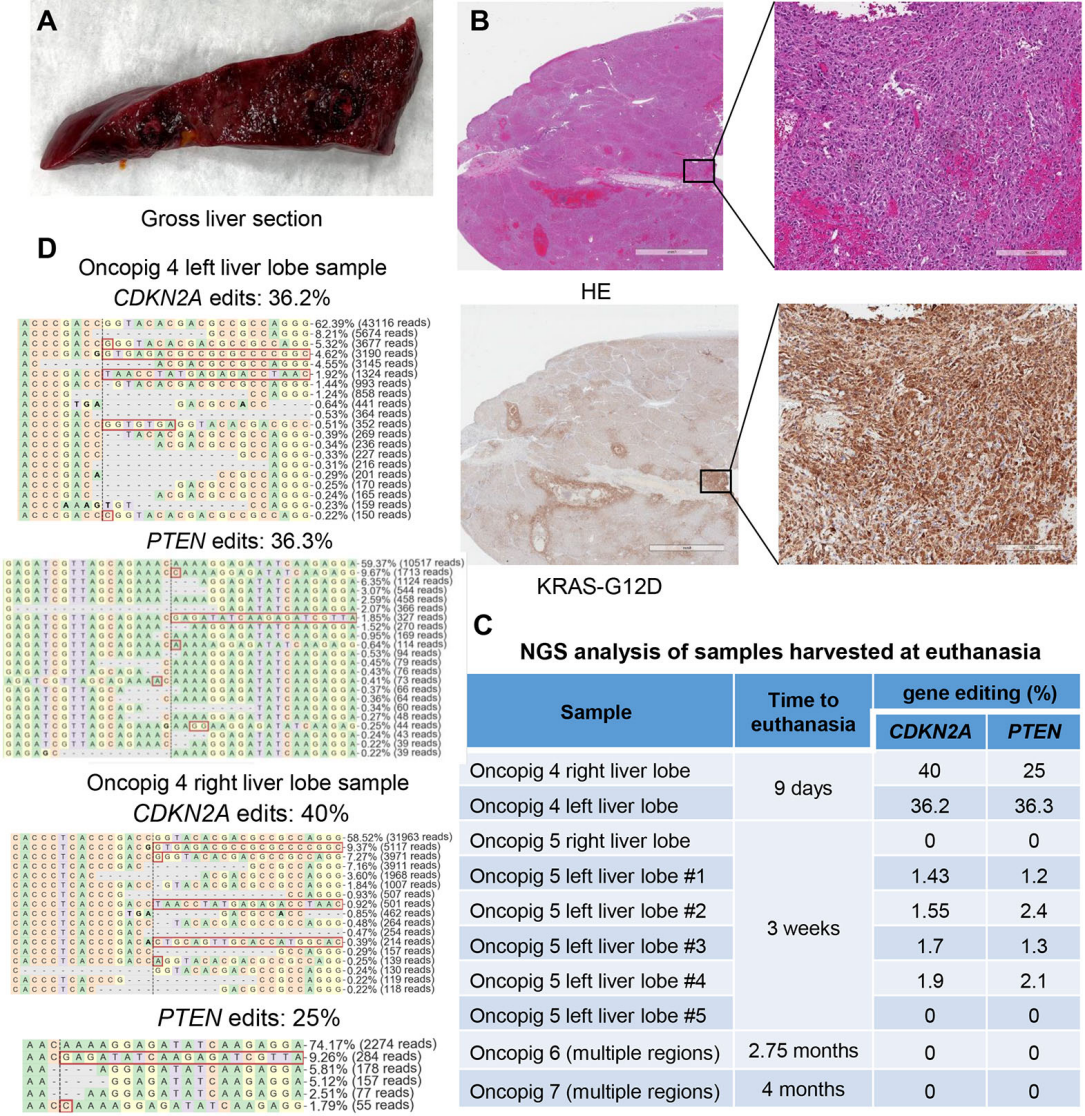
**Portal vein injection of autologous CRISPR-edited cells results in the development of Oncopig multifocal intrahepatic tumors that regress over time.** (A) Representative image of gross liver section harvested from Oncopig 4 at 9 days post-injection of autologous CRISPR-edited HCC cells via the portal vein. (B) Representative images of liver sections stained with H&E (HE) and KRAS^G12D^ demonstrate engraftment of injected HCC cells in the liver. Scale bars: 4 mm and 200 μm (insets). (C) Targeted NGS analysis detects *PTEN* and *CDKN2A* edits in liver sections from Oncopigs following portal vein injection of autologous CRISPR-edited cells. Engrafted cells with CRISPR edits are highest in liver sections harvested at earlier time points; CRISPR edits are not detected in liver sections collected at 2.5-4 months post-injection. (D) Reads detected by targeted NGS analysis in Oncopig liver sections following portal vein injections mapped to the reference sequences. The percentages of reads of each sequence are shown on the right. Dashed lines indicate the predicted Cas9 cleavage position; red boxes show insertions; dashes indicate deleted bases.

To develop multifocal HCC modeling advanced-stage HCC, another approach was used for cell delivery, entailing cell injection via the portal vein. Hepatocytes were isolated from Oncopigs (*n*=4) and exposed to Cre recombinase, followed by CRISPR KO of *PTEN* and *CDKN2A*. Predominance of CRISPR-induced indels in the cell pools was validated by Sanger sequencing analysis (Fig. S8). Liver fibrosis was either not induced (Oncopigs 4 and 5) or performed with a longer interval of nearly 4 months prior to cell injections (Oncopigs 6 and 7) to minimize the potential for increased tumor-associated inflammation due to performing the intrahepatic ethanol/ethiodized oil procedure in close temporal proximity to the cell injections (Table 1). Autologous cells were injected under fluoroscopic guidance into the left portal vein (Oncopigs 4 and 5) or the main portal vein (Oncopigs 6 and 7). Ultrasound imaging revealed the occurrence of portal vein thrombosis in Oncopigs 4 and 5, likely due to the injection of a concentrated cell suspension into smaller vein branches. This led to complications necessitating euthanasia of Oncopigs 4 and 5 at 9 days or 3 weeks post-injection, respectively (Table 1). Histological analysis revealed multifocal HCC in both pigs (Fig. 6A,B), with neoplastic cells forming effacing nodules, along with significant immune cell infiltration, including eosinophils. The neoplastic cells displayed strong cytoplasmic reactivity to KRAS^G12D^ IHC staining. Various abnormalities were also observed, including bile duct proliferation, loss of bile duct polarity, pseudogland formation, hemorrhage and calcification. Genomic analysis by targeted NGS identified *PTEN* and *CDKN2A* edits (25-40%) in Oncopig 4 liver sections, but less frequent edits in Oncopig 5 liver sections (0-2.1%) (Fig. 6C,D; Fig. S9).

To avoid portal vein thrombosis, Oncopigs 6 and 7 were injected with a less concentrated cell suspension into the main portal vein. Portal vein pressure was monitored during the injection and maintained within a range of 2 mmHg throughout the procedure. This adjustment successfully resulted in avoidance of portal vein thrombosis, confirmed by ultrasound imaging at 1 week post-injection. Importantly, multiple hypoattenuating masses measuring 1.5-2.0 cm each were identified in Oncopig 6 by CT done at 6 weeks post-injection. However, subsequent CT scan done at 11 weeks did not reveal any masses, suggesting tumor regression over time. Oncopig 7 did not develop detectable masses, as confirmed by CT at 7 weeks and 4 months post-injection, and necropsy revealed no masses in both Oncopigs, but abnormalities such as stiffened white ducts were observed. Histological analysis showed biliary hyperplasia, cholangitis and cystic bile ducts surrounded by dense fibrosis, thick collagen bands and marked inflammation, with infiltrating neutrophils and macrophages (Fig. S10). Bands of edematous fibrosis containing numerous blood and lymphatic vessels, as well as foci of smooth muscle hypertrophy, were observed. In several sections, portal tracts were edematous and associated with moderate lymphoplasmacytic inflammation. KRAS^G12D^ IHC staining was negative in liver samples from Oncopigs 6 and 7 (Fig. S10). Consistently, NGS analysis of random liver regions in Oncopigs 6 and 7 did not identify *PTEN* or *CDKN2A* edits (Fig. 6C). Collectively, these findings demonstrate the potential for establishing multifocal HCC in Oncopigs via portal vein injection of autologous transformed cells, while highlighting critical procedural adjustments necessary to prevent complications and support tumor growth over extended time periods.

## DISCUSSION

Preclinical models with precise tumor mutations that mirror human HCC are crucial for evaluating novel precision medicine and investigating the functional significance of disease-related mutations, ultimately improving patient outcomes. Leveraging CRISPR/Cas9 gene editing, we developed a porcine orthotopic implantation HCC model with defined tumor mutations mimicking common alterations in human HCC. Porcine models offer substantial advantages owing to their significant anatomical, physiological, immunological, genetic and metabolic similarities to humans (Flisikowska et al., 2016). This resemblance enables them to effectively bridge the gap between murine studies and clinical applications. Moreover, their comparable size to humans facilitates the testing of devices and procedures not feasible to test in small-animal models such as mice. The size similarity is particularly beneficial for HCC models because it allows testing image-guided locoregional therapies such as intra-arterial therapies, which are widely used in clinical practice (Llovet et al., 2021a; Nurili et al., 2021). Although porcine models of HCC have been developed by chemical induction or genetic engineering (Gaba et al., 2020; Ho et al., 2018; Li et al., 2006; Mitchell et al., 2016; Nurili et al., 2021; Schachtschneider et al., 2017), chemically induced models do not allow precise tumor gene manipulations (Mitchell et al., 2016), and genetically engineered large animals are extremely costly and time consuming to produce (Perleberg et al., 2018). Our feasible approach addresses these limitations by leveraging an existing transgenic pig model and somatic CRISPR editing to provide a versatile platform of genetically modifiable HCC models in large animals, thereby advancing HCC translational research.

To the best of our knowledge, the porcine HCC model described herein is the first immunocompetent large-animal model for HCC that allows the introduction of defined tumor mutations through somatic gene editing. This model serves as a promising tool valuable for evaluating novel therapeutic strategies, including LRTs and/or systemic therapies, and for advancing diagnostics and imaging approaches within the context of defined tumor driver mutations. Notably, our model closely mimics key aspects of human HCC. First, the tumors harbor gene mutations similar to those frequently observed in human HCC, with oncogenic mutations in *TP53* and *KRAS* induced by activation of the Oncopig transgene, and loss-of-function mutations in *PTEN* and *CDKN2A* induced by CRISPR/Cas9. Consistent with their established tumor suppressor roles in human HCC, PTEN and CDKN2A deficiencies increased porcine HCC cell proliferation and migration. Second, our model was developed concurrently with liver fibrosis, a comorbidity present in nearly 90% of HCC cases. Liver fibrosis was induced in Oncopigs by intrahepatic injection of ethanol mixed with ethiodized oil and oral alcohol administration. Incorporating disease comorbidities is critical for modeling unique tumor microenvironments and accurately assessing therapeutic responses. Third, characteristic HCC-associated changes frequently observed in human disease were observed in our model, including angiogenesis, vascular proliferation, hyperplasia, bile duct proliferation and excessive extracellular matrix (Brunt, 2012; Kim et al., 2020; Vij and Calderaro, 2021). Additionally, employing two different injection approaches enabled the development of both localized and multifocal tumors, representing distinct stages of human HCC.

Although our current study developed HCC tumors with *PTEN* and *CDKN2A* loss-of-function mutations, the system is readily adaptable to develop HCC with other mutational profiles in Oncopigs (Elkhadragy et al., 2020). Indeed, we recently demonstrated the development of Oncopig subcutaneous tumors harboring CRISPR-induced mutations in *ARID1A* and/or *AXIN1* (Elkhadragy et al., 2022). This versatility can extend beyond HCC models, offering potential applications for developing other cancer models with clinically relevant tumor mutations. Furthermore, this study builds the foundation for developing orthotopic implantation HCC models in wild-type pigs through *in vitro* transformation of hepatocytes, enabling the introduction of diverse gene mutations without reliance on transgenic pigs. Broadening porcine cancer models to capture a wider array of clinically relevant mutational profiles will advance *in vivo* investigations of tumor biology and therapeutic interventions.

Despite its significance, this study has several limitations, including the small number of animals used, inconsistent timing of blood and imaging assessments, and tumor necrosis and regression occurring at later time points. These limitations highlight the need for future studies with larger animal cohorts to further validate and characterize the model. Additional controls are necessary to delineate the effects of liver fibrosis, different scaffold materials and varying cell injection numbers on Oncopig HCC progression. Typically, orthotopic HCC models involve the injection of cells mixed with a scaffold material, such as Matrigel. In this study, we used porcine-derived gelatin sponge to avoid potential immune rejection associated with mouse-derived Matrigel. However, gelatin sponge injections without cells were not included as controls, and other scaffold materials, such as synthetic hydrogels, have not been tested. Future investigations could also investigate whether injecting smaller cell numbers results in tumor formation with reduced necrosis and inflammation. Constraints such as ethical considerations, logistical complexities and high costs typically preclude the use of larger numbers in large-animal studies. These logistical complexities can be alleviated by utilizing centralized facilities dedicated to pig studies, which streamline resources and reduce cost duplication. Additionally, establishing comprehensive, shared data repositories containing porcine blood reference values, validated antibodies and CRISPR gRNAs, as well as histological and imaging characteristics, can significantly decrease the costs and time associated with individual researcher optimization efforts. Given the outbred nature of pigs, we adopted an autologous implantation approach for tumor development, necessitating the injection of gene-edited HCC cells in the same pig from which the hepatocytes were isolated. Employing an autologous approach substantially increases workload, the timeline for model development and associated costs, contributing to the small number of animal subjects. Logistic challenges with large animals also contributed to the inconsistent imaging timings and oral ethanol timing and duration. Notably, a robust anti-tumor immune response was observed in tumors developed by percutaneous liver or portal vein injections, consistent with findings in other Oncopig cancer models, including pancreatic and lung cancers (Mondal et al., 2023; Overgaard et al., 2018; Ghosn et al., 2023). Although this immune response recapitulates a subset of human patients and allows studying anti-tumor immune response, it is also a primary contributor to necrosis and tumor regression, limiting the model's suitability for long-term efficacy studies (Elkhadragy et al., 2024). Our findings suggest that this model is best suited for short-term drug testing, ideally within 1-2 weeks post-injection.

The utility of these porcine models can be broadened by potential refinements. For instance, employing a viral vector expressing Cre recombinase and CRISPR components under the control of a hepatocyte-specific promoter would enable the *in vivo* development of genetically defined HCC tumors, eliminating the need for hepatocyte isolation and *in vitro* cell culture. This approach has been successfully applied in genetically engineered mouse models (Sanchez-Rivera et al., 2014) but has yet to be applied in porcine models. Alternative approaches that can decrease the workload and costs associated with autologous implantation include immunosuppression and/or matching donor and recipient major histocompatibility complex (MHC) haplotypes and blood types to allow tumor growth from allogeneic cell injections. Additional studies modeling other HCC comorbidities, such as metabolic syndrome, concurrent with Oncopig tumor induction would provide valuable expansion for this model system.

In conclusion, this study demonstrates the feasibility of developing a large-animal model of HCC with tailored tumor mutations through somatic cell CRISPR editing, laying the foundation for future studies aimed at further validating and characterizing a panel of personalized large-animal models of cancer. These models hold immense promise for advancing precision treatment strategies for HCC by identifying optimal therapeutic targets and prognostic biomarkers, paving the way for transformational progress toward improved stratified HCC therapeutics.

## MATERIALS AND METHODS

### Animals

All animal procedures were conducted at the University of Illinois at Chicago (UIC) or the University of Illinois Urbana-Champaign and were approved by the University of Illinois Institutional Animal Care and Use Committee. Seven male Oncopigs (age 1.3-2.5 months at the start of the experiments) heterozygous for the transgene construct were used in this study (Table 1). Male pigs were used in this study based on epidemiological data and findings from mouse models, which demonstrate a higher predisposition for HCC in males (He et al., 2015; Heindryckx et al., 2009). Results from 11 normal control Oncopigs, collected as part of other studies, were included in this paper as follows: histological analysis of liver tissue collected from an age-matched female control Oncopig is presented in Fig. 5C; serum laboratory values from ten age-matched normal control Oncopigs (four male and six female Oncopigs) are included in Fig. S4. Guidelines for reporting animal research have been followed consistent with Animals in Research: Reporting *In vivo* Experiments (ARRIVE) (Kilkenny et al., 2010). A completed ARRIVE guidelines checklist is included in Table S1.

### Development and culturing of Oncopig HCC cells

Porcine HCC cells were developed as previously described (Gaba et al., 2020; Elkhadragy et al., 2020). Briefly, hepatocytes were isolated from Oncopigs by collagenase digestion of surgically resected liver sections (for Oncopigs 1, 4, 5, 6 and 7) or liver biopsies obtained under ultrasound guidance (for Oncopigs 2 and 3). Isolated hepatocytes were exposed to adenovirus expressing Cre recombinase (Ad5CMVCre-eGFP; Viral Vector Core Facility, University of Iowa, Iowa City, IA, USA) to induce transgene expression. Porcine HCC cells were maintained in Dulbecco's modified Eagle medium (DMEM) supplemented with 10% fetal bovine serum (FBS) and 1% penicillin-streptomycin. Transgene expression was confirmed by RT-PCR (Fig. S11). All culture media and supplements were purchased from Gibco/Thermo Fisher Scientific, Waltham, MA, USA.

### CRISPR/Cas9 gene editing

gRNAs were designed using CRISPOR as previously described (Concordet and Haeussler, 2018; Elkhadragy et al., 2020), and the AltR^®^ CRISPR/Cas9 system (IDT Corporation, Hillside, IL, USA) was used for gene editing. Each gRNA was synthesized by incubating equimolar ratios of CRISPR RNA (crRNA) (sequences in Table S2, synthesized by IDT Corporation) and trans-activating CRISPR RNA (tracrRNA) (1072532; IDT Corporation) at 95°C for 5 min followed by cooling to room temperature. Purified *Streptococcus pyogenes* Cas9 nuclease (1081060; IDT Corporation) diluted in Opti-MEM (31985062; Gibco/Thermo Fisher Scientific) was then combined with the gRNA at equimolar ratio to form a ribonucleoprotein (RNP) complex. Porcine HCC cells were transfected with equimolar ratios of PTEN RNP and CDKN2A RNP using Lipofectamine CRISPRMAX transfection reagent (CMAX00003; Invitrogen, Carlsbad, CA, USA) following the manufacturer's instructions.

### Analysis of gene editing in porcine HCC cells

Genomic DNA was extracted from cells using QuickExtract DNA Extraction Solution (QE09050; Lucigen, Middleton, WI, USA) as previously described (Elkhadragy et al., 2020). The genomic locus that flanks the Cas9 target site was amplified by PCR for 28 cycles using the primers listed in Table S2. PCR products were then provided to the UIC Genome Research Core for Sanger sequencing. The gene editing efficiency was calculated using Inference of CRISPR Edits (ICE) software, which quantifies the frequency of sequences containing insertions and deletions (indels).

### Cell proliferation assay

Cell proliferation was determined by MTS assay using a CellTiter 96^®^ AQueous One Solution Cell Proliferation Assay Kit (G3580; Promega, Madison, WI, USA), following the manufacturer's instructions, using a BioTek 800 TS Absorbance Reader (BioTek, Winooski, VT, USA).

### Cell migration assay

Cell migration was analyzed using a modified two-chamber transwell system (353097; Corning, Corning, NY, USA). Cells were detached by trypsin/EDTA, washed once with serum-free medium and then resuspended in serum-free medium. Complete culture medium with 10% FBS was added to each bottom well. In total, 25,000 cells were added in each transwell insert and allowed to migrate for 18 h in a 37°C cell incubator, then the cells in the upper surface of the transwell were removed using cotton swabs. The migrated cells attached on the undersurface were fixed with 4% paraformaldehyde for 15 min and stained with Crystal Violet solution (0.5% in water) for 10 min. Migrated cells were then photographed and counted. The quantitated migration ability was presented as the number of migrated cells per field.

### Liver fibrosis induction in Oncopigs

Liver fibrosis was induced by oral administration of ethanol solution and intrahepatic injection of ethanol–ethiodized oil mixture as shown in Table 1. Oncopigs 1, 3, 6 and 7 received hepatic transarterial infusion of 0.75 ml/kg of a 1:3 v/v emulsified mixture of absolute ethanol and ethiodized oil, and Oncopig 2 received hepatic transarterial infusion of 0.75 ml/kg of 1:2 v/v ethanol–ethiodized oil emulsion, as previously described (Gaba et al., 2018). Oral ethanol solution was introduced as a mixture of Cherry Kool-Aid (Kraft Heinz, Chicago, IL, USA) with Swine BlueLite^®^ (TechMix) with an incremental ramp of 2.5% (v/v) per day until 15% (v/v) ethanol was well tolerated. The daily volume of oral ethanol solution administered to pigs represented 30% of normal daily caloric intake. Water was available to Oncopigs *ad libitum*. Ethanol feeding was withheld on days that Oncopigs underwent procedures and for an additional recovery day. Oral ethanol administration was initiated before cell injections and continued until euthanasia for Oncopigs 1, 2, and 3, or for a total of 5.5 months for Oncopigs 6 and 7, as shown in Table 1. Oncopigs 4 and 5 were not subjected to liver fibrosis induction.

### Ultrasound-guided percutaneous liver injections

Sixty million porcine HCC cells were washed twice with PBS, re-suspended in 1 ml PBS and autologously injected into the liver parenchyma of anesthetized Oncopigs as previously described (Nurili et al., 2021). Briefly, the cells were drawn into a 3 ml syringe and mixed with small pieces of gelatin sponge (Surgifoam; Ethicon, Somerville, NJ, USA) by passing four to five times through a three-way stopcock (Cook Medical, Bloomington, IN, USA). This mixture was injected into the hepatic parenchyma using a 16-gauge needle with stylet (Echogenic Co-axial Introducer Needle; Argon Medical Devices, Wheeling, IL, USA), which was advanced percutaneously under ultrasound guidance into the liver. Each Oncopig (1, 2, 3) received autologous cell injections at four sites. The number of cells injected in each site was based on previous studies (Nurili et al., 2021). Injection sites were selected to be as far apart as possible, technically feasible and safe to access, and deep enough to avoid leakage of injected material into the peritoneum.

### Portal vein cell injections

With Oncopigs under general anesthesia, an intrahepatic portal vein branch was identified under ultrasound guidance. Access to the portal vein was gained through a percutaneous needle and 0.018″ guidewire. A 3F introducer was inserted, and iodinated contrast medium was injected to confirm portal vein access. In Oncopigs 4 and 5, 150 million autologous porcine HCC cells suspended in 15 ml saline were injected via the left portal vein over 6.5 min (for Oncopig 4) or 7.5 min (for Oncopig 5), followed by washing with 3-4 ml saline. In Oncopigs 6 and 7, 70 million autologous porcine HCC cells suspended in 7 ml saline were injected via the main portal vein over 6 min, followed by washing with saline and insertion of two metallic coils (Nester Embolization Coil; Cook, Bloomington, IN, USA) for track hemostasis and to prevent cell backflow. Portal vein pressure was measured at baseline and every 2 min during the injection procedure in Oncopigs 6 and 7.

### Imaging and harvesting of intrahepatic tumors

Ultrasound imaging and CT scans were performed to monitor tumor growth. CT protocol included non-contrast, arterial phase at 30-35 s, portal venous phase at 60-70 s, and delayed phase at 180 s after 120-150 ml of iodinated contrast injected intravenously at a rate of 4-5 ml/s. Tumor volume was calculated using the following equation:
(Eqn1)

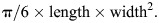


Ultrasound-guided biopsies were obtained from tumors and normal liver at the timepoints shown in Table 1. Intrahepatic masses harvested at euthanasia were transected; one half was stored in 10% formalin for histological processing and the other half was flash frozen in liquid nitrogen and stored at −80°C for genomic analysis.

### Analysis of gene editing in intrahepatic masses by NGS

Genomic DNA was extracted from intrahepatic masses and tissues using a DNeasy Blood and Tissue Kit (69504; Qiagen, Hilden, Germany). The genomic locus that flanks the Cas9 target site was amplified by PCR for 28 cycles using the primers listed in Table S2. These primers contain NGS adaptors used to add additional adaptor sequences and barcodes as part of the Fluidigm library preparation. PCR products were then provided to the UIC Genome Research Core for library preparation and sequencing. Briefly, a second PCR was done to attach Fluidigm barcode and NGS adaptor sequences to the amplicons generated. Samples were then pooled and targeted NGS was performed using a MiSeq instrument (2 x150 kit, Illumina, San Diego, CA, USA) following the manufacturer's instructions. The gene editing efficiency was calculated using the CRISPRESSO2 alignment tool (Pinello et al., 2016), which quantifies the frequency of sequences containing indels.

### Histology

Intrahepatic mass and liver samples were processed for H&E staining and IHC staining of KRAS^G12D^ using anti- Ras^G12D^ antibody (26036; NewEast Biosciences, King of Prussia, PA, USA) and arginase-1 using anti-Arginase antibody (ab91279; Abcam, Cambridge, UK). Liver sections were also stained with trichrome stain. Whole slides were scanned using a Hamamatsu Nanozoomer scanner (Hamamatsu Photonics, Hamamatsu City Japan), and digital images were visualized with Aperio ImageScope software (Leica, Lincolnshire, IL, USA).

### Statistical analysis

Data were expressed as mean±s.d. in proliferation assays and as mean±s.e. in migration assays. Statistical significance was determined by unpaired two-tailed Student's *t-*test in migration assays or by two-way analysis of variance (ANOVA) in cell proliferation assays. *P*<0.05 was considered statistically significant.

## Supplementary Material

10.1242/dmm.052079_sup1 Supplementary information
