# Supplementary material for: Development of a genetically tailored implantation hepatocellular carcinoma model in Oncopigs by somatic cell CRISPR editing
Source: Dis Model Mech. 2025 Jan 29;18(1):dmm052079. doi: 10.1242/dmm.052079 (PMC11810043; doi:10.1242/dmm.052079)
Supplement: Supplementary information [file dmm-18-052079-s1.pdf]

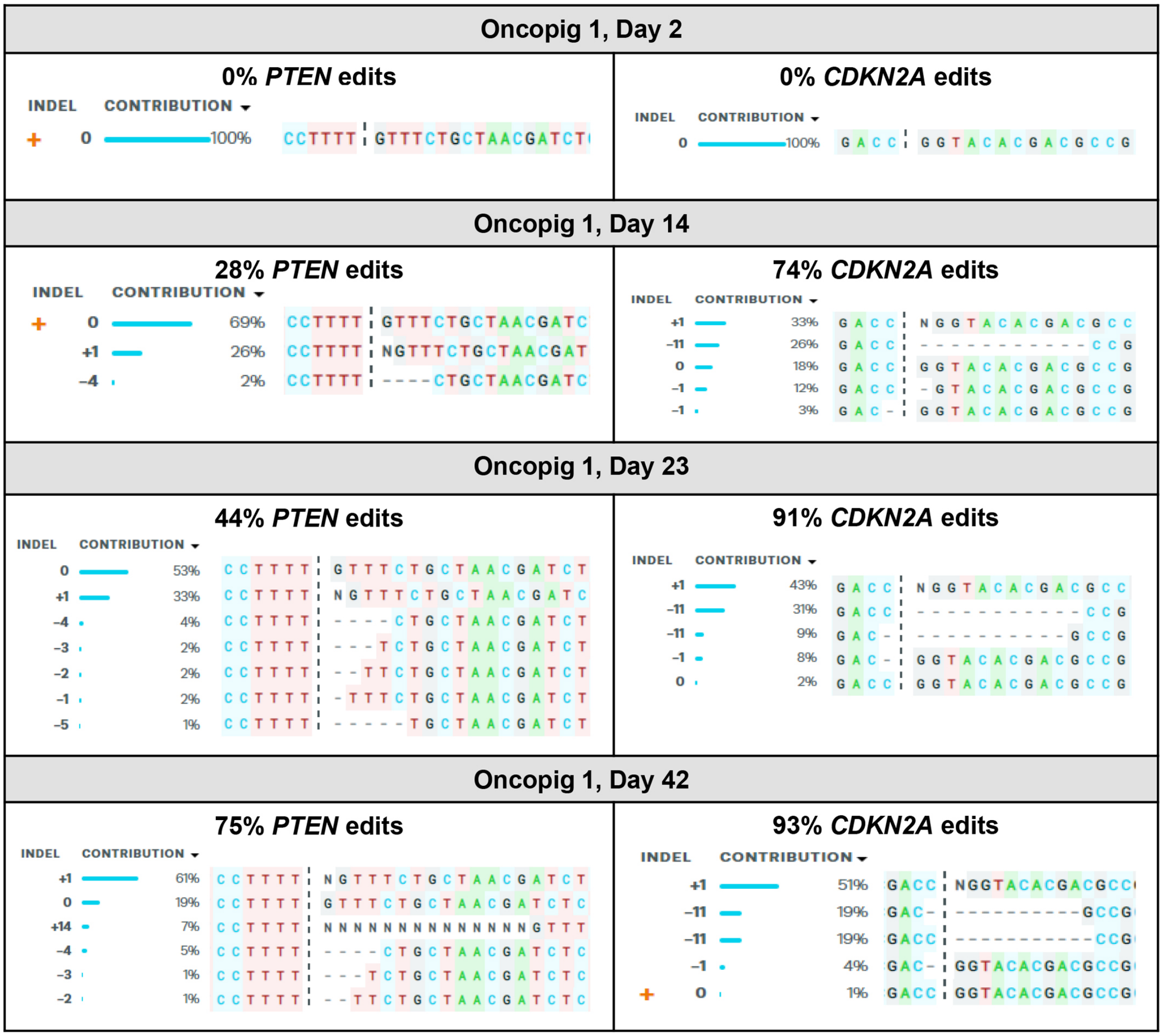

Fig. S1. Enrichment of cells with *PTEN*^{KO} and *CDKN2A*^{KO} in Oncopig 1 HCC cells cultured *in vitro* over time. Oncopig 1 HCC cells were transfected with RNPs comprising Cas9 and gRNAs targeting *PTEN* and *CDKN2A* and DNA was extracted from cells in culture at several time points. Sanger sequencing (ICE) analysis depicts an enrichment of cells with KO in *PTEN* and *CDKN2A* over time. Type and frequency of *PTEN* or *CDKN2A* indels mapped to the reference sequence. Dashed line, predicted Cas9 cleavage position; N, nucleotide insertion; dash, deleted base.

Disease Models & Mechanisms • Supplementary information

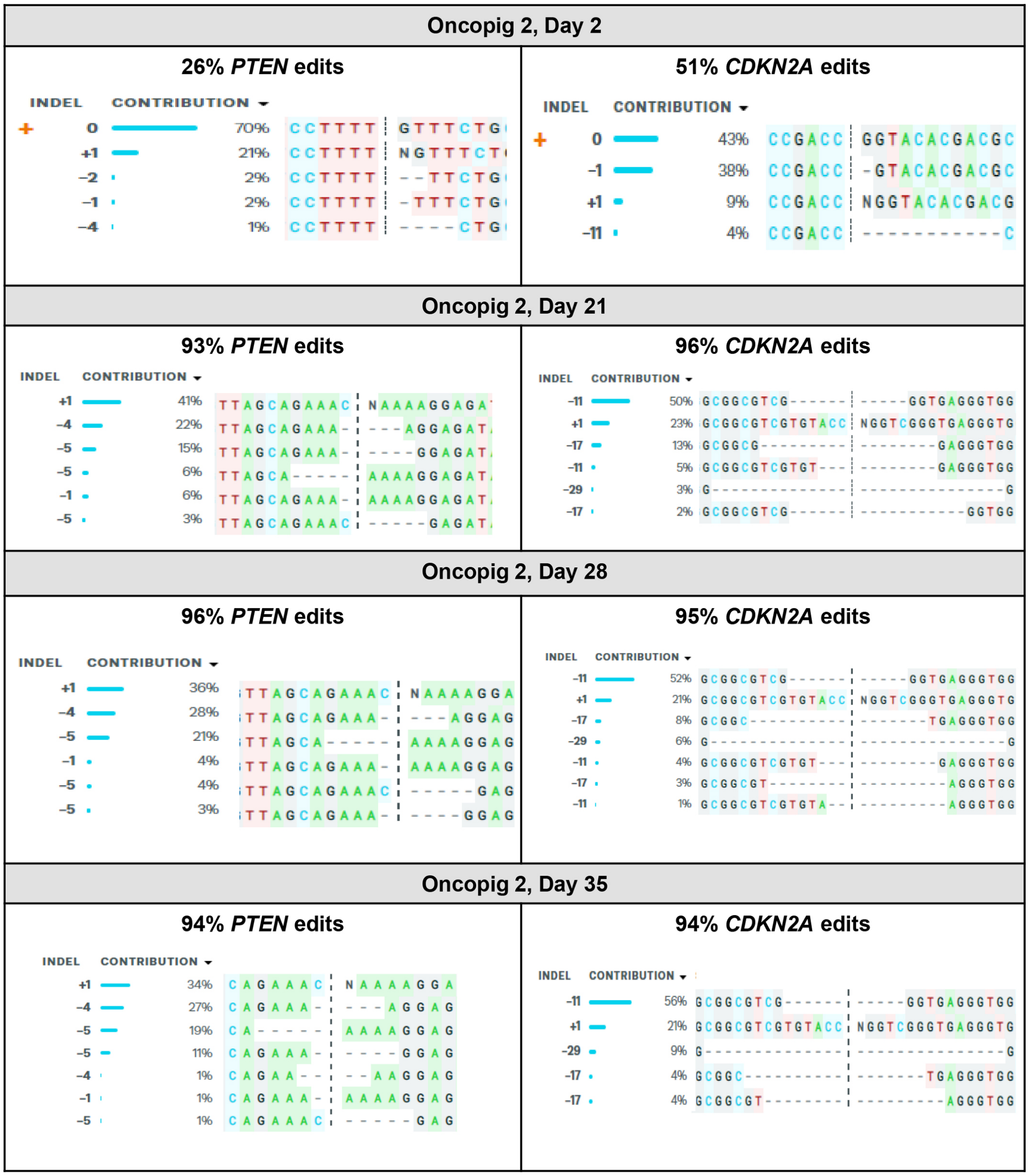

Fig. S2. Enrichment of cells with *PTEN*^{KO} and *CDKN2A*^{KO} in Oncopig 2 HCC cells cultured *in vitro* over time. Oncopig 2 HCC cells were transfected with RNPs comprising Cas9 and gRNAs targeting *PTEN* and *CDKN2A* and DNA was extracted from cells in culture at several time points. Sanger sequencing (ICE) analysis depicts an enrichment of cells with KO in *PTEN* and *CDKN2A* over time. Type and frequency of *PTEN* or *CDKN2A* indels mapped to the reference sequence. Dashed line, predicted Cas9 cleavage position; N, nucleotide insertion; dash, deleted base.

Disease Models & Mechanisms • Supplementary information

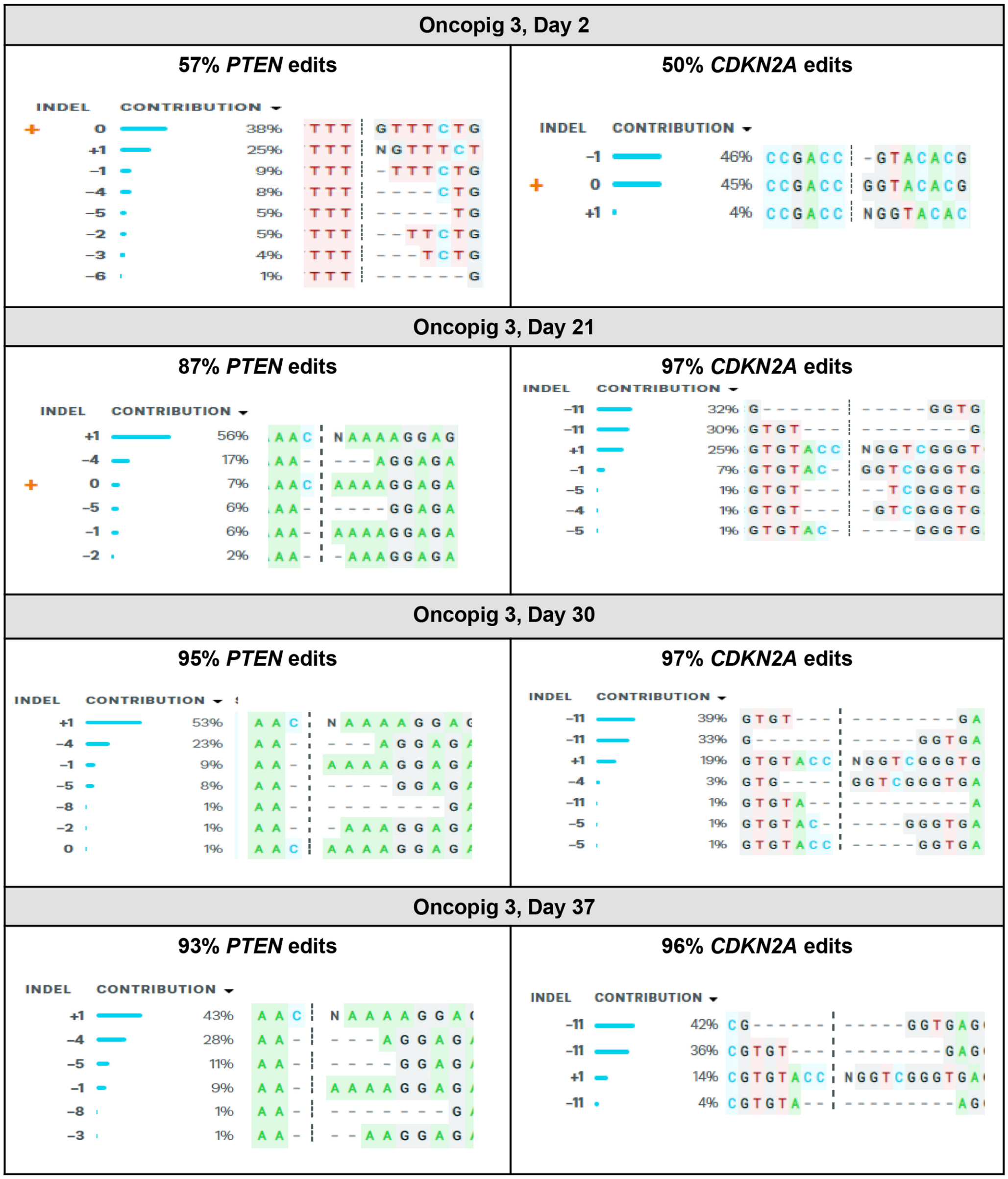

Fig. S3. Enrichment of cells with *PTEN*^{KO} and *CDKN2A*^{KO} in Oncopig 3 HCC cells cultured *in vitro* over time. Oncopig 3 HCC cells were transfected with RNPs comprising Cas9 and gRNAs targeting *PTEN* and *CDKN2A* and DNA was extracted from cells in culture at several time points. Sanger sequencing (ICE) analysis depicts an enrichment of cells with KO in *PTEN* and *CDKN2A* over time. Type and frequency of *PTEN* or *CDKN2A* indels mapped to the reference sequence. Dashed line, predicted Cas9 cleavage position; N, nucleotide insertion; dash, deleted base.

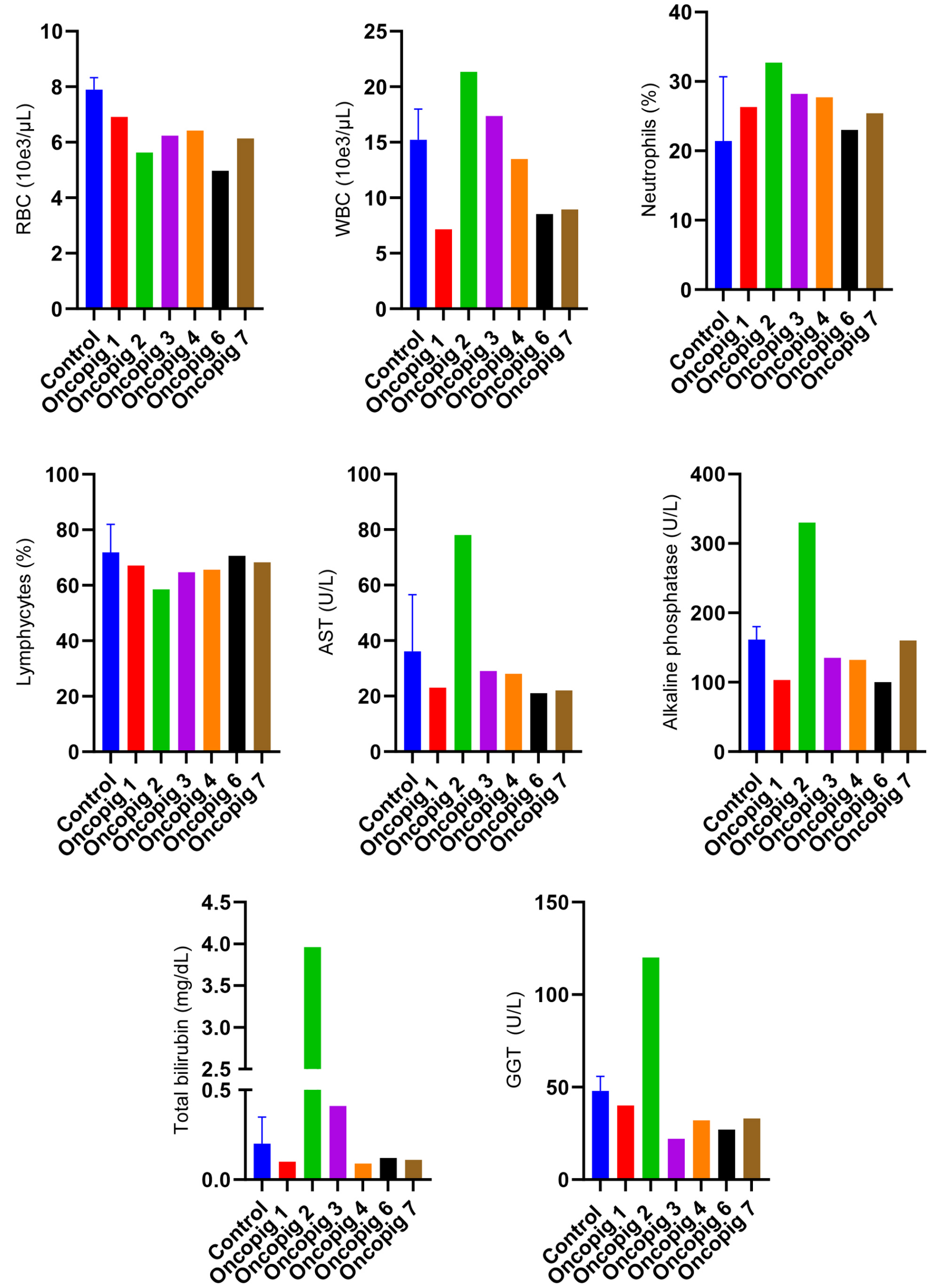

Fig. S4. Hematological and liver function analyses in experimental Oncopigs serum after tumor induction compared to control age-matched Oncopigs.

Disease Models & Mechanisms • Supplementary information

Blood was collected from Oncopigs at the following time points post cell injection: Oncopig 1 (2-months post-injection), Oncopigs 2, 3, 4, and 6 (2-weeks post-injection), and Oncopig 7 (1-month post-injection). Blood was also collected from age-matched control Oncopigs (4 male Oncopigs and 6 female Oncopigs). The following parameters were analyzed: red blood cell (RBC) count, white blood cell (WBC) count, neutrophils (%), lymphocytes (%), aspartate transferase (AST, U/L), alkaline phosphatase (U/L), total bilirubin (mg/dL), and gamma-glutamyl transferase (GGT, U/L). Mean ± S.D. is depicted for control Oncopig lab values in the bar graphs.

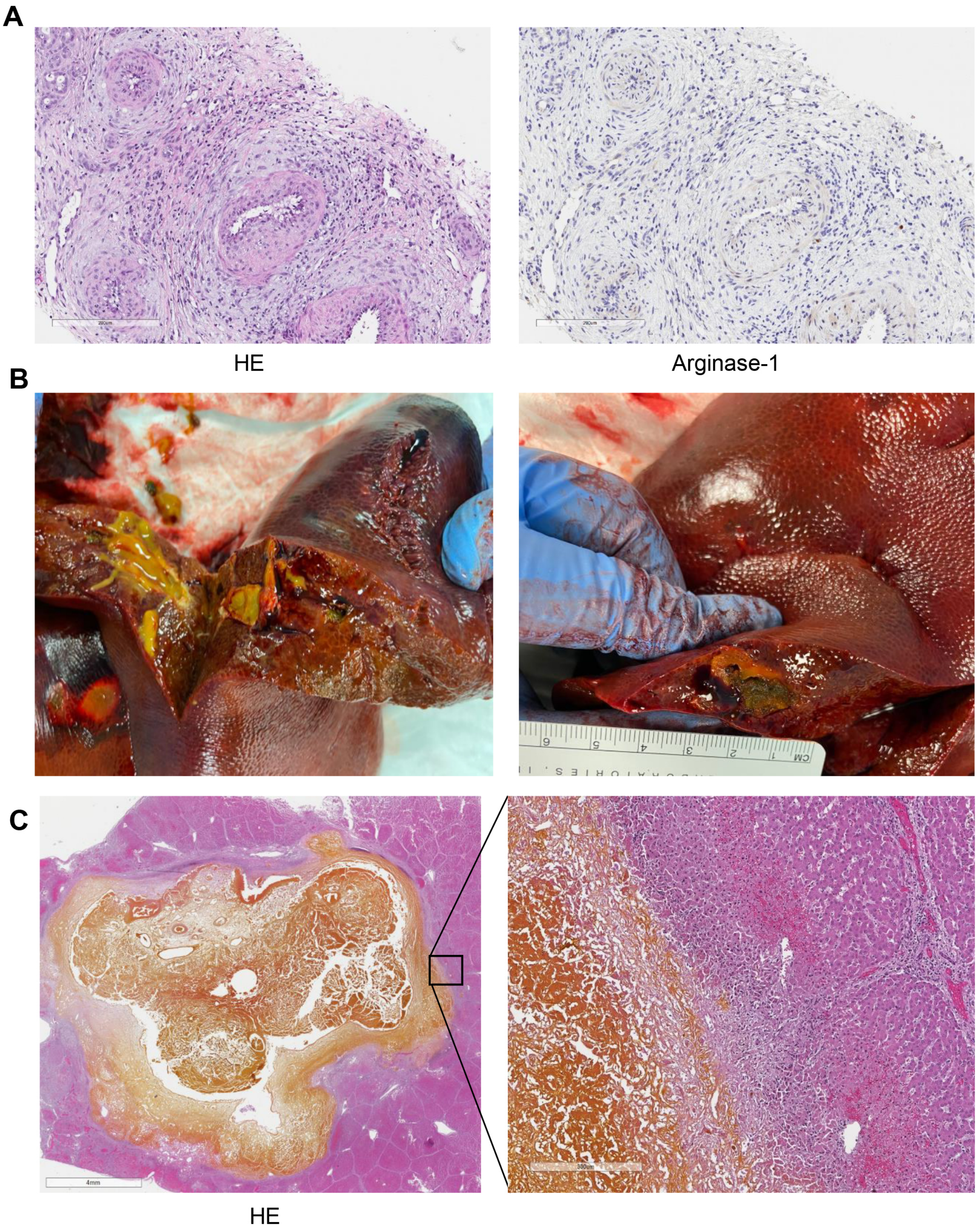

Disease Models & Mechanisms • Supplementary information

Fig. S5. Oncopig 2 develops biliary stricture adverse events.

(A) Ultrasound-guided biopsies were obtained from Oncopiog 2 heterogeneous region in the left liver lobe at 2-weeks post-injection. H&E staining shows bile duct proliferation, arterial vascular proliferation, and presence of fibrous tissue. Negative Arginase-1 staining of the liver biopsy sample. Scale bar, 200 µm. (B) Representative images of gross liver sections harvested from Oncopig 2 at euthanasia done 1-month post-injection. Excessive bile flow and necrotic tissues were observed in the liver. (C) H&E staining of Oncopig 2 liver section reveals cholestasis and necrosis. Scale bar, 5 mm, inset scale bar, 300 µm

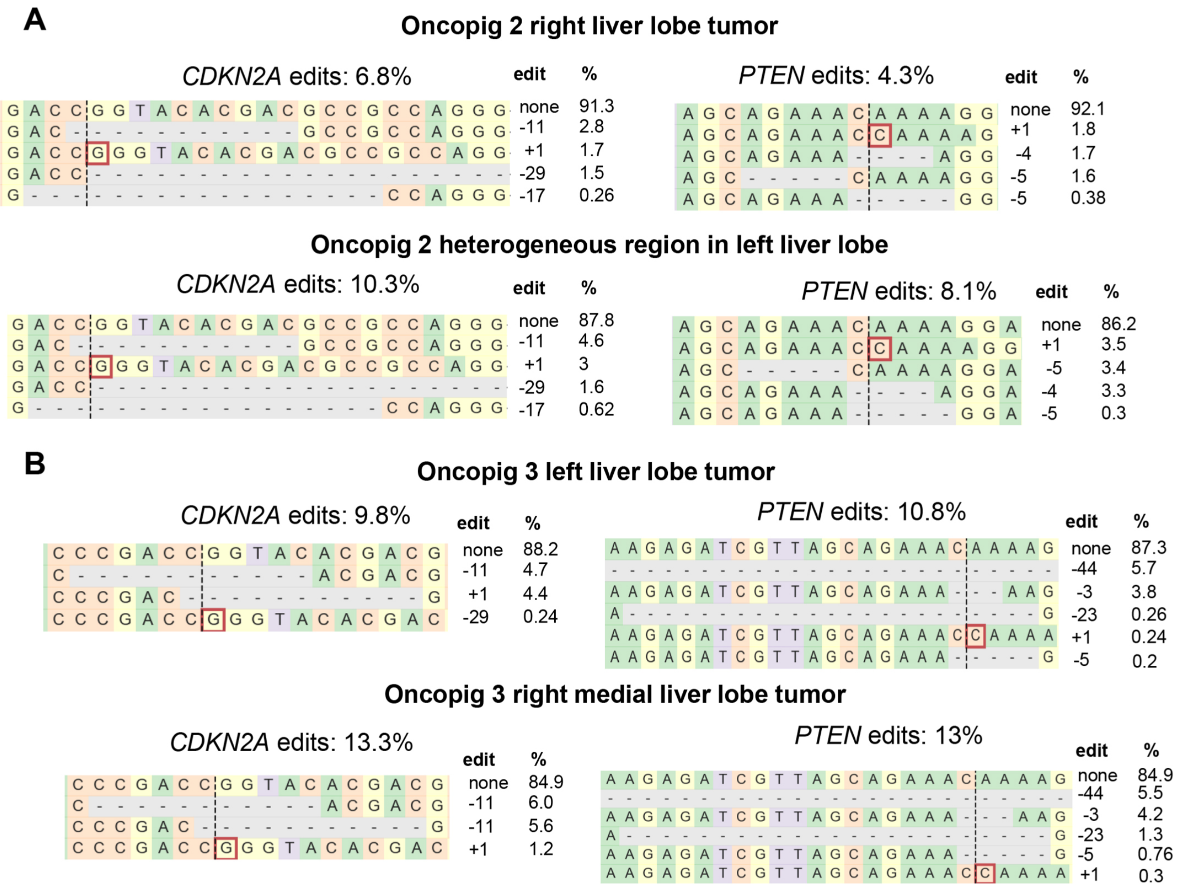

Fig. S6. CRISPR induced edits in *PTEN* and *CDKN2A* detected in intrahepatic tumor biopsies by NGS analysis.

(A) Reads detected by targeted NGS analysis of biopsies obtained under ultrasound-guidance from Oncopig 2 right liver lobe tumor and left lobe heterogenous region mapped to the reference sequences. (B) Reads detected by targeted NGS analysis of biopsies obtained under ultrasound-guidance from Oncopig 3 left liver lobe tumor and right medial liver lobe tumor mapped to the reference sequences. The percentage of reads of each sequence are shown on the right. Dashed line, predicted Cas9 cleavage position; red box, insertion; dash, deleted base.

Disease Models & Mechanisms • Supplementary information

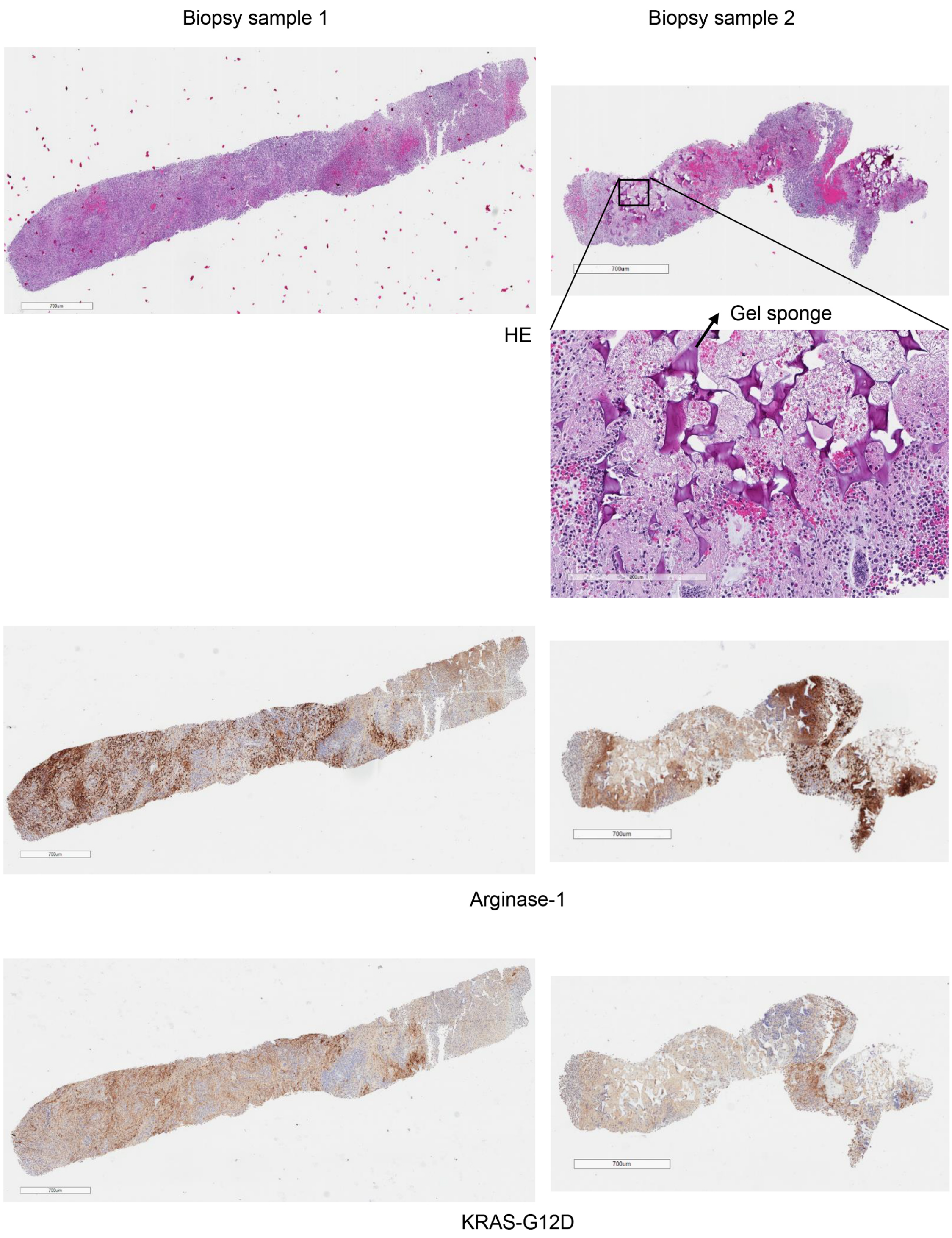

Disease Models & Mechanisms • Supplementary information

Fig. S7. Variability among Oncopig intrahepatic tumor biopsies. Low magnification images of two biopsy samples taken from Oncopig 3 lateral right liver lobe tumor, stained with H&E, Arginase-1, or KRASG12D reveal variability both within each biopsy and between the samples. High magnification of H&E stained biopsy sample shows gel sponge surrounding necrotic areas. Scale bar, 700 μm, inset scale bar, 200 μm

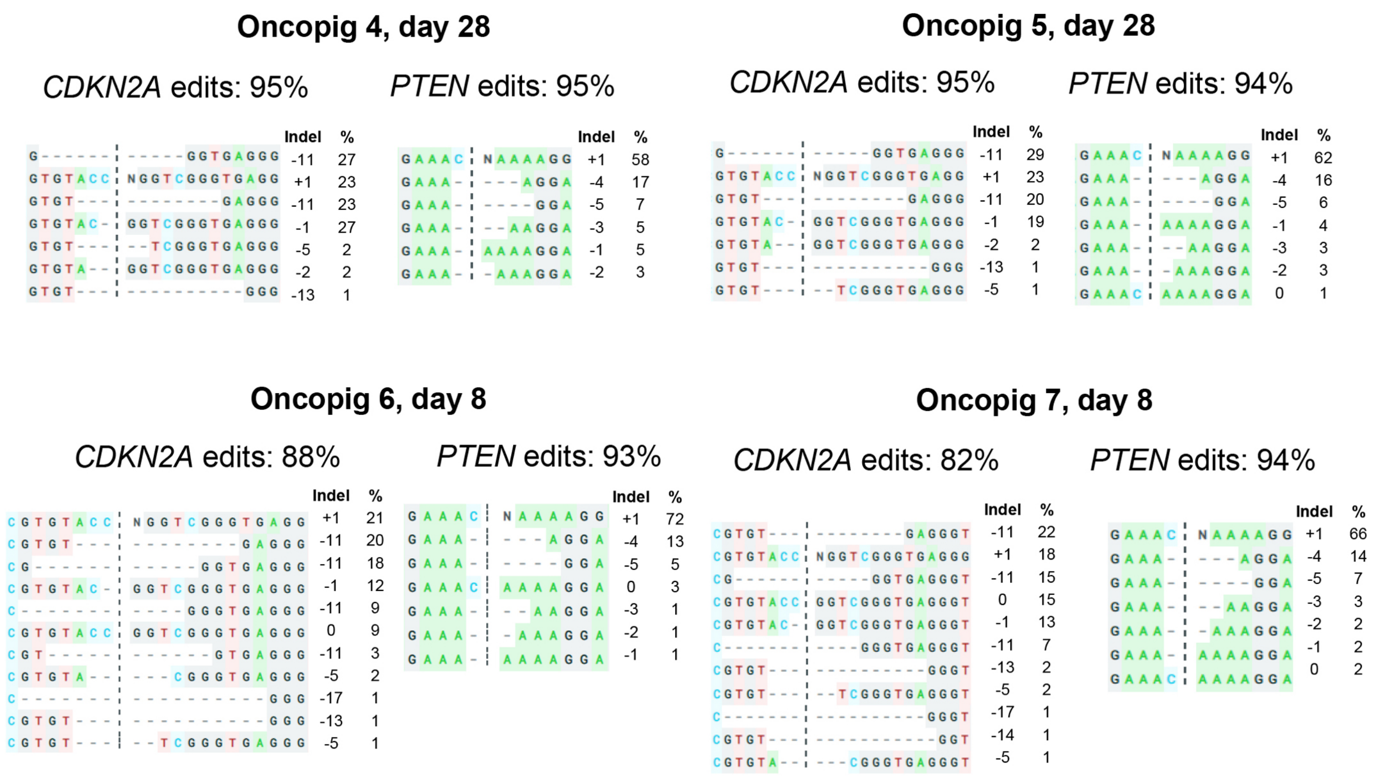

Fig. S8. CRISPR induced edits in *PTEN* and *CDKN2A* in HCC cells injected autologously into Oncopigs via the portal vein.

Porcine HCC cells developed from four individual Oncopigs (Oncopig 4-7) were transfected with gRNAs against *PTEN* and *CDKN2A* simultaneously. Cells were cultured *in vitro* and DNA was obtained at specific time points and subjected to Sanger sequencing analysis of CRISPR-induced indels. Percent indels, type, and frequency of *PTEN* or *CDKN2A* indels mapped to the reference sequence are shown. Dashed line, predicted Cas9 cleavage position; N, nucleotide insertion; dash, deleted base.

Disease Models & Mechanisms • Supplementary information

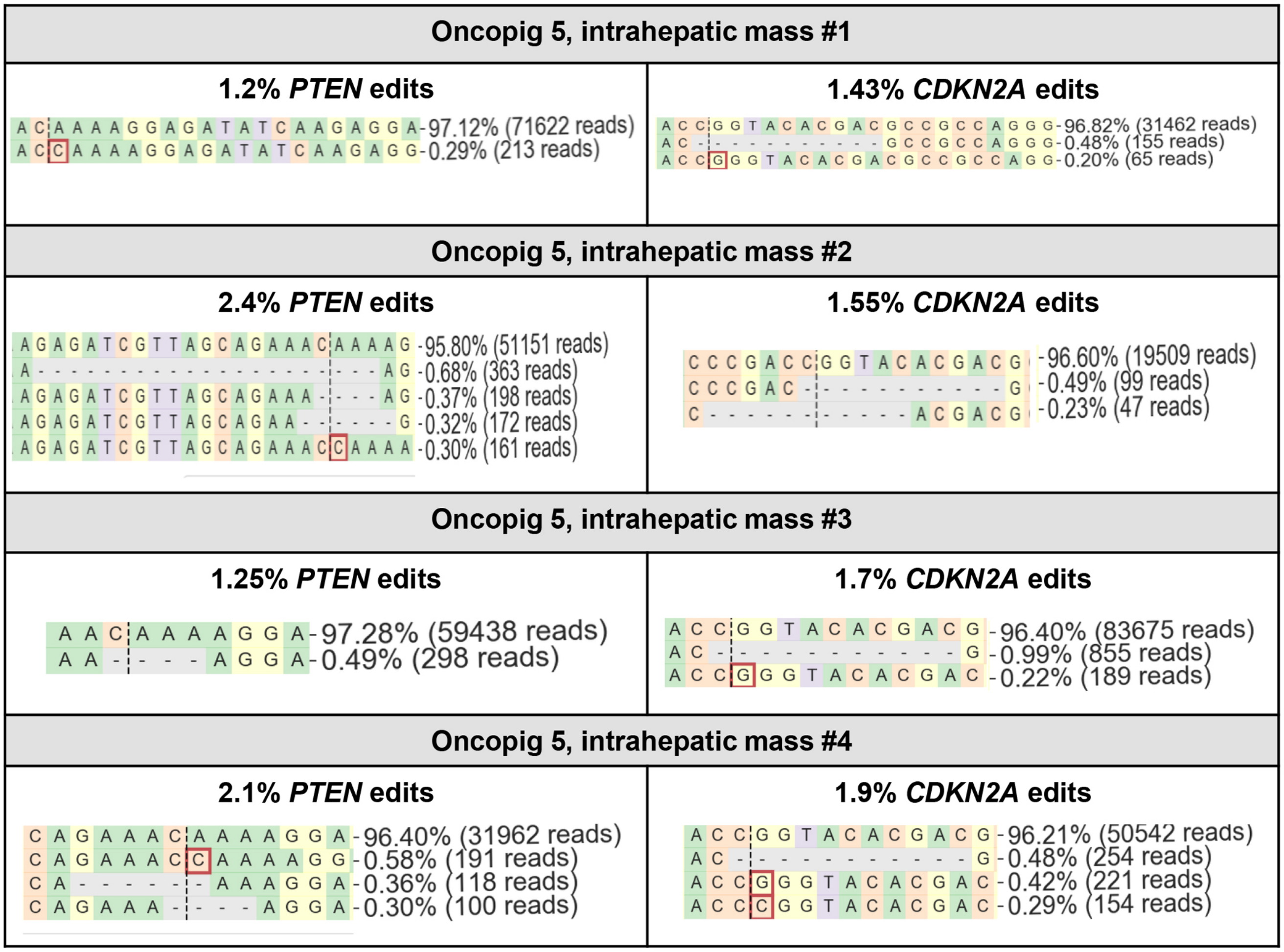

Fig. S9. NGS analysis of *CDKN2A* and *PTEN* confirms presence of CRISPR-edited cells in intrahepatic masses and tissues at 3 weeks after portal vein injection of autologous cells in Oncopigs.

Reads detected by targeted NGS analysis of intrahepatic masses obtained from Oncopig 5 at euthanasia mapped to the reference sequences. The percentage of reads of each sequence are shown on the right. Dashed line, predicted Cas9 cleavage position; red box, insertion; dash, deleted base.

Disease Models & Mechanisms • Supplementary information

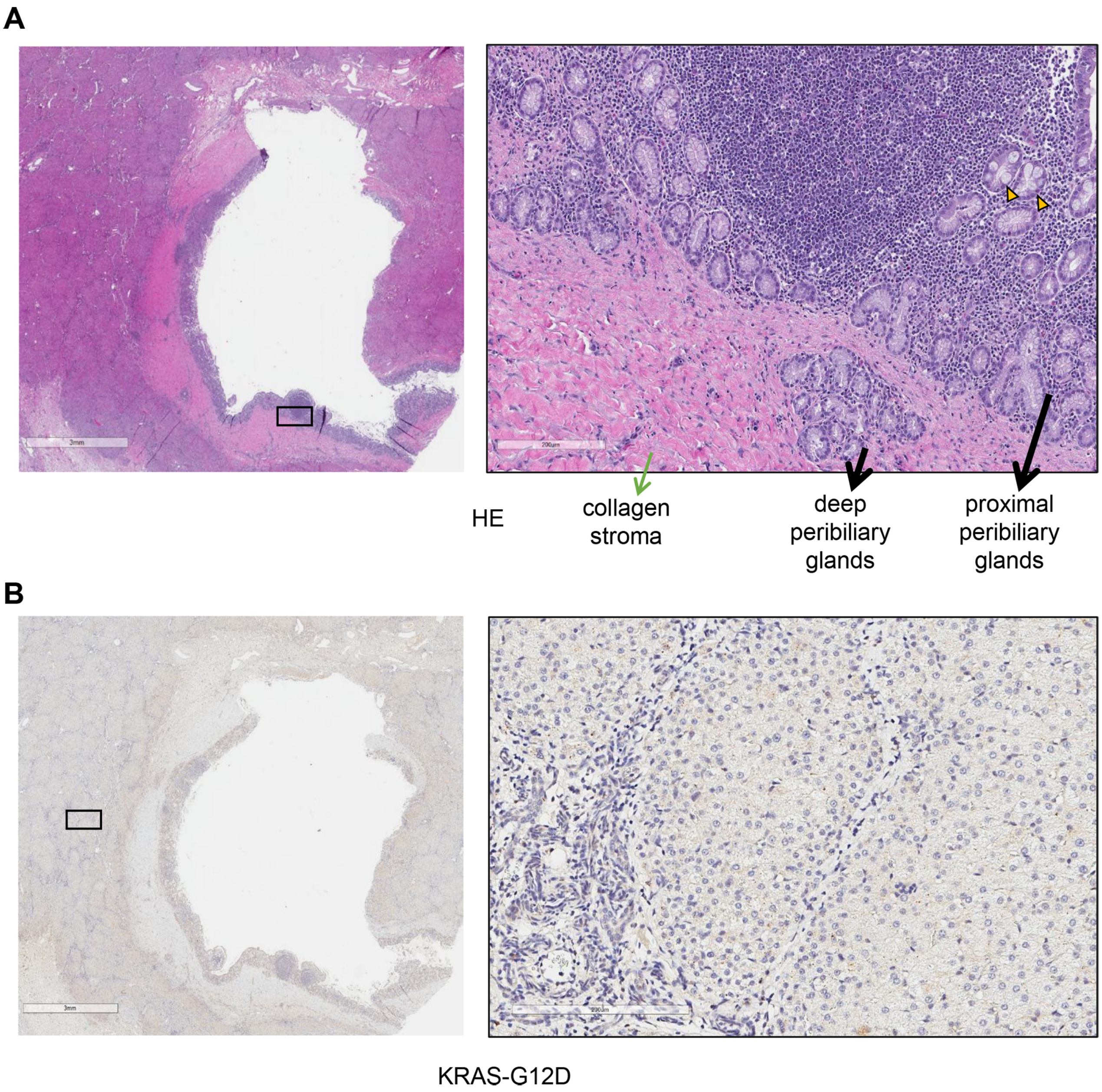

Fig. S10. Histological analysis of liver sections from Oncopigs 6 and 7 reveal biliary hyperplasia with marked inflammation.

Representative image for Oncopig 6 liver section stained with H&E and KRAS^{G12D}. (A) H&E stained section shows a large bile duct lined with simple columnar epithelium with goblet cell metaplasia (yellow arrowhead) and abundant peribiliary glands (black arrows) surrounded by dense chronic immune cell infiltrates and collagenous stroma (green arrow). (B) KRAS^{G12D} staining is negative. Scale bar, 3 mm, inset scale bar, 200 µm.

A

RT-PCR Primers
KRAS^{G12D} primers:
Forward: TTGTACAGCTAGCTGCTGAAAATGACTGAATAT
Reverse: ATTCTCGAGCGGTTACATAATTATACAC
TP53^{R167H} primers:
Forward: TGGCTCTCCTCAAGCGTATT
Reverse: ATTTTCATCCAGCCAGTTCG

B

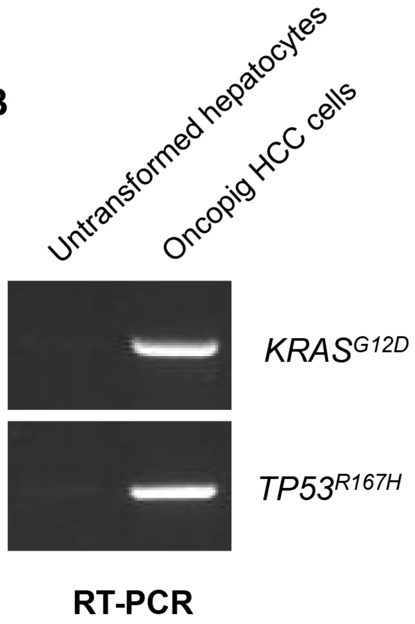

Fig. S11. Confirmation of transgene expression in Oncopig HCC cells by RT-PCR. (A) Sequences of primers used for RT-PCR. (B) Representative image for gel electrophoresis of RT-PCR products confirming the expression of KRAS^{G12D} and TP53^{R167H} Oncopig transgenes following exposure to Adenovirus expressing Cre recombinase.

Disease Models & Mechanisms • Supplementary information

Table S1. Completed ARRIVE guidelines checklist.

The ARRIVE guidelines 2.0: author checklist

The ARRIVE Essential 10

These items are the basic minimum to include in a manuscript. Without this information, readers and reviewers cannot assess the reliability of the findings.

Item		Recommendation	Section/line number, or reason for not reporting
**Study design**	1	For each experiment, provide brief details of study design including:	
		a. The groups being compared, including control groups. If no control group has been used, the rationale should be stated.	Methods/ Animals
		b. The experimental unit (e.g. a single animal, litter, or cage of animals).	Methods/ Animals
**Sample size**	2	a. Specify the exact number of experimental units allocated to each group, and the total number in each experiment. Also indicate the total number of animals used.	Methods/ Animals
		b. Explain how the sample size was decided. Provide details of any *a priori* sample size calculation, if done.	Sample size calculation not done prior to study.
**Inclusion and exclusion criteria**	3	a. Describe any criteria used for including and excluding animals (or experimental units) during the experiment, and data points during the analysis. Specify if these criteria were established *a priori*. If no criteria were set, state this explicitly.	No criteria were set.
		b. For each experimental group, report any animals, experimental units or data points not included in the analysis and explain why. If there were no exclusions, state so.	No exclusions.
		c. For each analysis, report the exact value of *n* in each experimental group.	Table 1
**Randomisation**	4	a. State whether randomisation was used to allocate experimental units to control and treatment groups. If done, provide the method used to generate the randomisation sequence.	No randomization has been performed.
		b. Describe the strategy used to minimise potential confounders such as the order of treatments and measurements, or animal/cage location. If confounders were not controlled, state this explicitly.	Confounders were not controlled.
**Blinding**	5	Describe who was aware of the group allocation at the different stages of the experiment (during the allocation, the conduct of the experiment, the outcome assessment, and the data analysis).	LE, MJC, LRJ, KMS, RCG
**Outcome measures**	6	a. Clearly define all outcome measures assessed (e.g. cell death, molecular markers, or behavioural changes).	Liver tumor development
		b. For hypothesis-testing studies, specify the primary outcome measure, i.e. the outcome measure that was used to determine the sample size.	N/A
**Statistical methods**	7	a. Provide details of the statistical methods used for each analysis, including software used.	Methods
		b. Describe any methods used to assess whether the data met the assumptions of the statistical approach, and what was done if the assumptions were not met.	N/A
**Experimental animals**	8	a. Provide species-appropriate details of the animals used, including species, strain and substrain, sex, age or developmental stage, and, if relevant, weight.	Methods/Animals
		b. Provide further relevant information on the provenance of animals, health/immune status, genetic modification status, genotype, and any previous procedures.	Methods/Animals
**Experimental procedures**	9	For each experimental group, including controls, describe the procedures in enough detail to allow others to replicate them, including:	
		a. What was done, how it was done and what was used.	Methods
		b. When and how often.	Methods
		c. Where (including detail of any acclimatisation periods).	Methods
		d. Why (provide rationale for procedures).	Results
**Results**	10	For each experiment conducted, including independent replications, report:	
		a. Summary/descriptive statistics for each experimental group, with a measure of variability where applicable (e.g. mean and SD, or median and range).	Results, Table 1
		b. If applicable, the effect size with a confidence interval.	N/A

Disease Models & Mechanisms • Supplementary information

Table S2. Oligonucleotides used in the study.

**crRNAs**	
**Control crRNA**	5'- GGCGCGTATAGTCGCGCGTA -3'
***PTEN* crRNA#1**	5'- GCTAACGATCTCTTTGATGA -3'
***PTEN* crRNA#2**	5'- AGATCGTTAGCAGAAACAAA -3'
***CDKN2A* crRNA#1**	5'- TGGCGGCGTCGTGTACCGGT -3'
***CDKN2A* crRNA#2**	5'- CGGCGCAGACCCCAACTGCG -3'
**PCR primers for Sanger sequencing**	
**PTEN-For1**	5'- GAACGCCGGAGAGTTGGTCTC -3'
**PTEN-Rev1**	5'- GAGACCCCGTAGACAGACTAAGA -3'
**CDKN2A-For1**	5'- AGTGCAACGTTCAGGGTAACT -3'
**CDKN2A-Rev1**	5'- TCTCCAGAGTTTGAGCGAGC -3'
**PCR primers for targeted sequencing**	
**PTEN-For2**	5'-ACACTCACGACATGGTTCTACACATTTCCATCCTGCAGAAGAAGCC-3'
**PTEN-Rev2**	5'-TACGGTAGCAGAGACTTGGTCTACGTTATAAGAGCGAGTGACAGAAA-3'
**CDKN2A-For2**	5'-ACACTCACGACATGGTTCTACAATGATGATGGGCAGCACCC-3'
**CDKN2A-Rev2**	5'-TACGGTAGCAGAGACTTGGTCTCCTCTCCTCAGCCAGGTCG-3'

The shaded regions represent adaptor sequences for barcode attachment.

Disease Models & Mechanisms • Supplementary information
